# From 0D-complex to 3D-MOF: changing the antimicrobial activity of zinc(II) *via* reaction with aminocinnamic acids

**DOI:** 10.3389/fchem.2024.1430457

**Published:** 2024-07-08

**Authors:** Simone d’Agostino, Laura Macchietti, Raymond J. Turner, Fabrizia Grepioni

**Affiliations:** ^1^ Dipartimento di Chimica “Giacomo Ciamician”, Università di Bologna, Bologna, Italy; ^2^ Department of Biological Sciences, University of Calgary, Calgary, AB, Canada

**Keywords:** MOFs, antimicrobial activity, zinc (II) complexes, aminocinnamic acid, solid state structure, mechanochemistry

## Abstract

Combining zinc nitrate with 3- and/or 4- aminocinnamic acid (3-ACA and 4-ACA, respectively) leads to the formation of the 0D complex [Zn(4-AC)_2_(H_2_O)_2_], the 1D coordination polymer [Zn(3-AC)(4-AC)], and the 2D and 3D MOFs [Zn(3-AC)_2_]∙2H_2_O and [Zn(4-AC)_2_]∙H_2_O, respectively. These compounds result from the deprotonation of the acid molecules, with the resulting 3- and 4-aminocinnamate anions serving as bidentate terminal or bridging ligands. All solids were fully characterized *via* single crystal and powder X-ray diffraction and thermal techniques. Given the mild antimicrobial properties of cinnamic acid derivatives and the antibacterial nature of the metal cation, these compounds were assessed and demonstrated very good planktonic cell killing as well as inhibition of biofilm growth against *Pseudomonas aeruginosa, Escherichia coli, and Staphylococcus aureus*.

## 1 Introduction

The emergence of antimicrobial resistance poses a formidable threat to global public health (Kibwana et al., 2024). Infections caused by pathogenic microorganisms (such as bacteria, fungi, and viruses) are increasingly becoming the primary cause of morbidity and mortality (Hall-Stoodley et al., 2004; Wei et al., 2019; Li et al., 2021). Traditional antibiotics, once hailed as miracle drugs, are increasingly falling prey to the adaptive strategies of bacteria, thus becoming less effective or even obsolete. As a result, the number of multidrug-resistant pathogens is in continuous growth (Levy and Bonnie, 2004; Streicher, 2021). To address this pressing issue, the scientific community is turning the attention to new formulations (Brown and Wright, 2016; Li et al., 2023).

According to the World Health Organization (WHO), the newly approved products demonstrate limited clinical advantages compared to existing treatments, since a significant portion of the antimicrobials currently in clinical development consists in variations of molecules already utilized in the market and recognized by bacteria, which exhibit multiple well-established resistance mechanisms (Levy and Bonnie, 2004; Brown and Wright, 2016; Streicher, 2021). Hence, it is crucial to expedite the development of novel antibacterial medications through innovative solutions.

Our goal in advancing the search for new antimicrobials involves the combination of crystal engineering principles (Braga et al., 2017; Shemchuk et al., 2020; Braga, 2023) with mechanochemical synthetic methods, as neat and liquid-assisted (LAG) co-grinding of solid reagents (Friščić et al., 2020; Michalchuk et al., 2021; Rautenberg et al., 2022). This also enables the synergistic effect of metal ions in complexes containing antimicrobials as organic ligands (Bolla et al., 2022). Metal salts that provide metal ion species in aqueous media are well known for their antimicrobial activity (Lemire et al., 2013).

**SCHEME 1:**
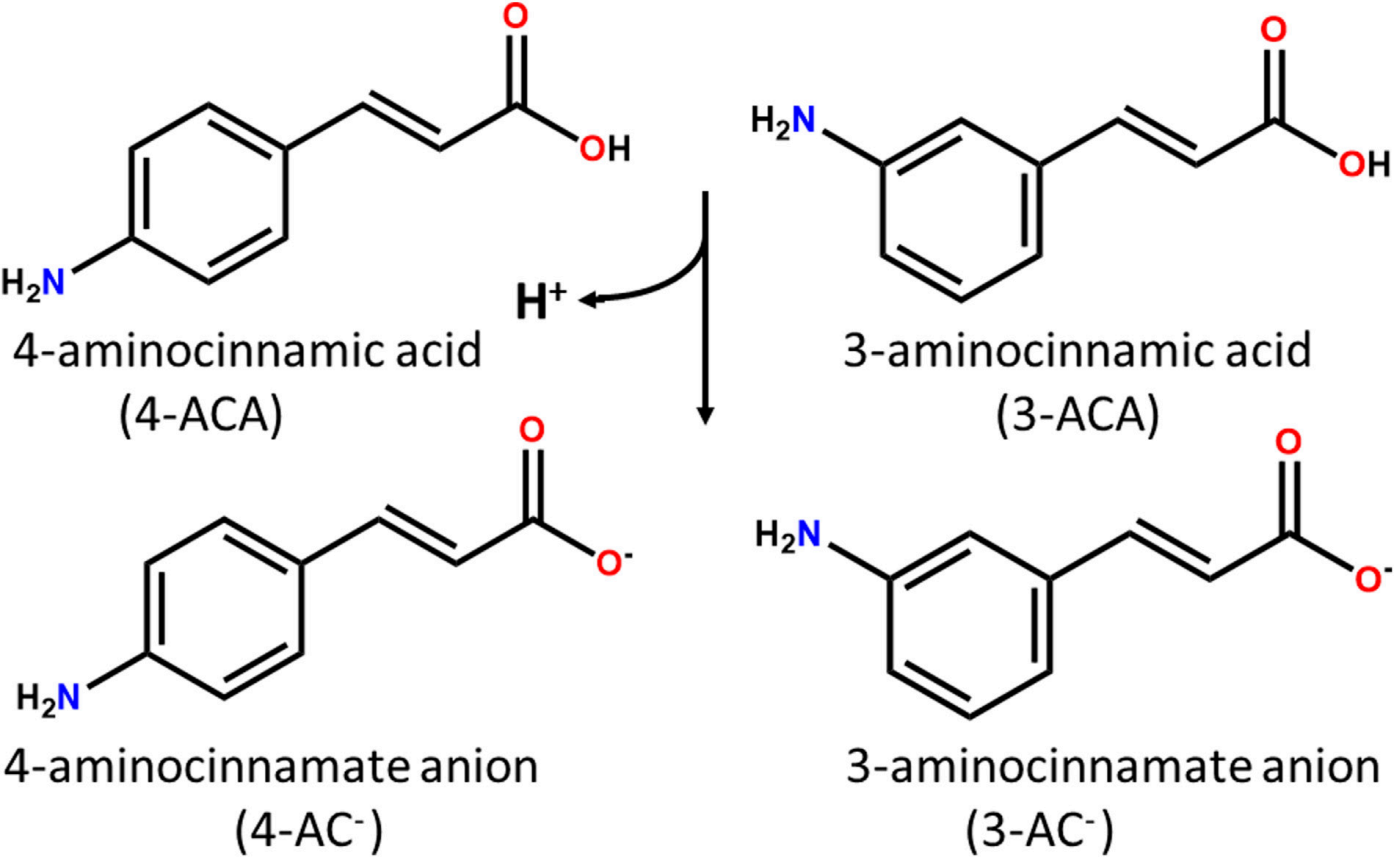
4- and 3- aminocinnamic acids (4-ACA and 3-ACA) and their deprotonated forms 4- and 3- aminocinnamate anions (4-AC^-^ and 3-AC^-^).

In this work we explore the potential antimicrobial properties of 3- and 4-aminocynnamic acids (Scheme 1) in combination with zinc(II), an *essential* element with antimicrobial activity.

The antimicrobial effects of zinc are established and operate through direct interactions with microbial membranes, inducing membrane instability and increased permeability (Ballin, 1974), or through interactions with nucleic acids and the inactivation of respiratory enzymes (Fang et al., 2006). Additionally, Zn^2+^ ions exhibit antimicrobial activity against a broad spectrum of microorganisms, including bacteria and fungi (Sirelkhatim et al., 2015; Hutchings et al., 2021). This versatility makes it a valuable component in the development of novel antimicrobial agents.

Plant based compounds and essential oils are well known for their antimicrobial activity. Of these cinnamaldehyde and cinnamic acid have gained increasing attention particularly as a potential food preservative (Sun et al., 2018; Pereira et al., 2021). Cinnamaldehyde was found to have antimicrobial and antibiofilm activity towards 6 WHO pathogens of concern on the same level as comparable plant compounds of Tea tree oil, Carvacrol and thymol (Pormohammad et al., 2022).

Cinnamic acid derivatives occur naturally in a number of plants and are central intermediates in the biosynthesis of a myriad of natural products (Vogt, 2010). Typically, they exhibit a strong absorption of UV rays, making them suitable components for formulating sunscreen products (Pattanaargson and Limphong, 2001; Pattanaargson et al., 2004; D’Agostino et al., 2020); besides, their antimicrobial properties have also been documented (Huang et al., 2009; Sova, 2012; Stefanović et al., 2015; Chukwuma et al., 2020).

Within the extensive array of cinnamic acid derivatives, the 3- and 4-amino derivatives, thanks to the presence of divergent -NH_2_ and -COO^-^ groups capable of coordinating metal ions, stand out as promising candidates for the construction of coordination polymers and Metal-Organic Frameworks (MOFs). These compounds have emerged as an extensive class of materials with a wide range of applications, spanning from energy and environment (Wang et al., 2017; Choi et al., 2019; Jiang et al., 2022; Yusuf et al., 2022) to catalysis (Pascanu et al., 2019; Alhumaimess, 2020; Khan et al., 2023; Obeso et al., 2023) recently they have been recognized as potential antimicrobial agents, and interest in their synthesis and solid state properties has been increasing (Bhardwaj et al., 2018; Li et al., 2021; Liu et al., 2021; Yan et al., 2022; Zhang et al., 2022).

MOFs, composed of metal ions linked and spaced by organic ligands possessing more than one coordinating group, are essentially porous coordination polymers, i.e., they possess potential voids, and are highly tunable solids. Their unique properties, such as high surface area and porosity, versatility, and, depending on the choice of organic spacers, biocompatibility, make them promising candidates for various biomedical applications, including sustained release of drugs and antimicrobial activity (Kaur et al., 2020; He et al., 2021; Maranescu and Visa, 2022; Yan et al., 2022). They have been shown to manifest synergistic effects via incorporation of multiple active components (Wang et al., 2022; Yusuf et al., 2022) they can also act as reservoirs and kill the pathogenic microorganisms through the slow and steady release of metal ions and drugs used as linkers (Azizabadi et al., 2021; Han et al., 2022).

This strategy may enhance the overall antimicrobial activity and reduce the likelihood of microbial resistance development and is the basis of our research. Given the mild antimicrobial properties of cinnamic acid derivatives and the antibacterial nature of the zinc(II) metal cation, combinations of these compounds into unique structures are expected to show additive or synergistic effects.

Several studies now have shown that metals with antimicrobial organics delivered as co-crystals or co-polymers show excellent potential as antimicrobial agents. Latest examples from our groups include the coordination of Ag, Cu, and Zn ions or salts with proflavine and kojic acid (Guerrini et al., 2022; Lekhan et al., 2022; Sun et al., 2023).

## 2 Materials and methods

### 2.1 Chemicals

Zn(NO_3_)_2_·6H_2_O was purchased from Sigma-Aldrich and used without further purification, 3-aminocinnammic acid and 4-aminocinnamic acid were bought from TCI and used after recrystallization (see below). Reagent grade solvents and Milli-Q water were used.

### 2.2 Recrystallization of 3- and 4-aminocinnamic acids

The compounds were purified *via* recrystallization from acetonitrile (MeCN), prior to their reaction with zinc(II). Recrystallization of 4-aminocinnamic acid (4-ACA) yielded pale yellow needle-like crystals, identified as the known form present in the Cambridge Structural Database (CSD refcode: BEZBAM) (D’Agostino et al., 2018), while *via* recrystallization of 3-aminocinnamic acid (3-AC) pale yellow needle-like crystals were obtained with some traces of white prismatic ones, further identified as the neutral and zwitterionic forms of the acid, respectively (see Supplementary Table S1; Supplementary Figures S1, S2).

### 2.3 Synthesis of [Zn(4-AC)_2_(H_2_O)2], [Zn(4-AC)_2_]∙H_2_O, [Zn(3-AC)_2_]∙2H_2_O, and [Zn(4-AC)(3-AC)]

#### 2.3.1 Mechanochemical synthesis

50 mg of 4-aminocinnamic acid (4-ACA) or 3-aminocinnamic acid (3-ACA), 13 mg of NaOH and 46.3 mg of Zn(NO_3_)_2_·6H_2_O (stoichiometric ratio: 1 : 1: 0.5) were placed in 10 mL steel jars and ball-milled in an Retsch MM200 for 60 min at 20 Hz. The milling products were isolated by washing with water to remove the NaNO_3_ formed as a byproduct. A modification of the above procedure, with a stoichiometric ratio 0.5 : 0.5: 1: 0.5, was employed for the synthesis of the mixed ligand compound. However, these mechanochemical syntheses yielded in most cases amorphous materials, and pure products were obtained only with 4-ACA. In the case of 3-ACA, or when a mixture of 3-ACA and 4-ACA was used, the reaction was often incomplete, and reproducibility was an issue.

#### 2.3.2 Solution synthesis

24 mg of NaOH were added to a suspension of 100 mg of 4-aminocinnamic acid (4-ACA) or 3-aminocinnamic acid (3-ACA) in *ca* 20 mL of H_2_O; the suspension was then left under stirring at RT for 10 min, affording a clear solution (pH = 7.5). Traces of unreacted aminocinnamic acid were filtered off by using a 5 mL syringe (InJ/Light) and an RC filter (Minisart, 200 μm). Adding 91.6 mg of Zn(NO_3_)_2_·6H_2_O to this solution resulted in a white powder suspension that was filtered under vacuum, washed with cold water (10 x 2 mL) and left to dry at RT in the dark, to afford polycrystalline samples suitable for powder XRD analysis. The mixed 1D coordination polymer [Zn(4-AC)(3-AC)] was obtained with the same procedure as before, but the reaction was carried out with 50 mg of 4-ACA and 50 mg of 3-ACA. Notably, the complex [Zn(4-AC)_2_(H_2_O)_2_] was obtained exclusively via solution methods by adjusting the pH to 6.5. Crystals suitable for XRD were grown in an NMR tube (d = 0.5 mm) by liquid diffusion of a Zn^2+^ solution layer through a solution of 3-aminocinnamate. All the attempts to grow [Zn(4-AC)(3-AC)] single crystals were unsuccessful, and structural solution could only be determined from powder XRD data (see below). For all compounds, yields were in the range 90%–95%. Elem. Anal. Calcd for [Zn(4-AC)_2_(H_2_O)_2_] (C_18_H_20_N_2_O_6_Zn): C, 50.78%; H, 4.74%; N, 6,58%. Found: C, 49.61%; H, 4.03%; N, 6.83%. Elem. Anal. Calcd for [Zn(4-AC)(3-AC)] (C_18_H_16_N_2_O_4_Zn): C, 55,48%; H, 4,14%; N, 7,19%. Found: C, 54,98%; H, 3.93%; N, 7.01%. Elem. Anal. Calcd for [Zn(3-AC)_2_]∙2H_2_O (C_18_H_20_N_2_O_6_Zn): C, 50.78%; H, 4.74%; N, 6,58%. Found: C, 50.01%; H, 4.53%; N, 6.97%. Elem. Anal. Calcd for [Zn(4-AC)_2_]∙H_2_O (C_18_H_18_N_2_O_5_Zn): C, 53,02%; H, 4.45%; N, 6, 87%. Found: C, 52.41%; H, 4.27%; N, 6.71%.

### 2.4 Single crystal X-ray diffraction (SCXRD)

Single crystal data for [Zn(4-AC)_2_(H_2_O)_2_], [Zn(4-AC)_2_]⋅H_2_O, and [Zn(3-AC)_2_]⋅2H_2_O were collected at RT on an Oxford X'Calibur S CCD diffractometer equipped with a graphite monochromator (Mo-Kα radiation, λ = 0.71073 Å). Reflection data were integrated and elaborated with the CrysAlisPro Software. The structures were solved with SHELXT (Sheldrick, 2015b) by intrinsic phasing and refined on F^2^ with SHELXL (Sheldrick, 2015a) implemented in the Olex2 software (Dolomanov et al., 2009) by full-matrix least squares refinement. H_OH_ and H_NH_ atoms were either directly located or, when not possible, added in calculated positions; H_CH_ atoms for all compounds were added in calculated positions and refined riding on their respective carbon atoms. All non-hydrogen atoms were refined anisotropically, and rigid-body restraints applied (Thorn et al., 2012). Data collection and refinement details are listed in Supplementary Table S1. The Mercury (Macrae et al., 2008) and Olex2 (Dolomanov et al., 2009) programs were used for molecular graphics and calculation of hydrogen bonding parameters, respectively.

### 2.5 Powder X-ray diffraction (PXRD)

Powder X-ray diffractograms for phase identification and thermal behavior analysis (VT-XRD) were collected in Bragg-Brentano geometry, in the 2θ range 5°–40°, on a Panalytical X’Pert PRO automated diffractometer equipped with an X'Celerator detector, using Cu Kα radiation without a monochromator (step size, 0.02°; time/step, 20 s; 0.04 rad soller; 40 mA × 40 kV). PXRD patterns, based on single crystal data collected in this work or retrieved from the CCDC (Groom et al., 2016), were calculated with the program Mercury (Macrae et al., 2008). In all cases the identity between polycrystalline samples and single crystals was verified by comparing experimental and calculated powder diffraction patterns (see Supplementary Figures S3, S4).

For the structural solution of [Zn(4-AC)(3-AC)] diffractograms (step size, 0.026°; time/step, 200 s; 0.02 rad soller; 40 mA × 40 kV) were collected on a Panalytical X’Pert PRO automated diffractometer equipped with a PIXcel detector in transmission geometry (capillary spinner), using Cu–Kα radiation without monochromator in the 2θ range 3°–65° (continuous scan mode, step size 0.0260°, counting time 889.70 s, soller slit 0.02, antiscatter slit ¼, divergence slit ¼, 40 mA × 40 kV). Five diffraction patterns were recorded and summed to enhance the signal-to-noise ratio. Powder diffraction data was analyzed with the software EXPO 2014 (Altomare et al., 2013), which is designed to analyze both monochromatic and non-monochromatic data. Selected peaks were chosen in the 2θ range 5°–50°, and unit cell constants were found using the algorithm N-TREOR09 (Altomare et al., 2000). The structure was solved by simulated annealing, employing fragments retrieved from the CCDC (Groom et al., 2016), and refined as a rigid body with the software EXPO 2014 (Altomare et al., 2013). An overall thermal parameter for all the atoms was adopted. See Supplementary Figure S5 for the difference plot pattern and Supplementary Table S1 for crystallographic details.

### 2.6 Thermogravimetric analysis (TGA)

TGA measurements were performed with a PerkinElmer TGA7 instrument in the temperature range 40°C–400°C, under a flow of N_2_ gas at a heating rate of 5.00°C min^-1^ (Supplementary Figure S6).

### 2.7 Attenuated total reflectance Fourier transform infra-red spectroscopy (ATR-FTIR)

ATR-FTIR spectra were obtained using a Bruker Alpha FT-IR spectrometer. Measurements were run for polycrystalline samples of 3-ACA and 4-ACA, and for the products obtained from solution: [Zn(4-AC)_2_(H_2_O)2], [Zn(4-AC)(3-AC)], [Zn(3-AC)_2_]⋅2H_2_O, and [Zn(4-AC)_2_]⋅H_2_O (see Supplementary Figure S7).

### 2.8 Antimicrobial assays

Antimicrobial efficacy of the compounds here presented was evaluated against type culture collection reference strains: *Pseudomonas aeruginosa* ATCC27853*, Escherichia coli* ATCC25922*, and Staphylococcus aureus* (ATCC25923). All microbial growth used Lysogeny broth (LB) prepared from milliQ water with 10 g/L NaCl (VWR International Co., Mississauga, ON, Canada), 5 g/L yeast extract (EMD Chemicals Inc., Darmstadt, Germany), and 10 g/L tryptone (VWR Chemicals LLC, Solon, OH, United States). The agar medium was prepared by adding 15 g/L bacteriological agar (VWR International LLC, Solon, OH, United States) to the LB prior to autoclaving. ZnSO_4_^.^H_2_O was obtained from Millipore Sigma (ON, Canada).

A standard Disk diffusion assay was performed as previously described (Guerrini et al., 2022). From an overnight culture 150 µL was spread on LB agar plates and left to dry at room temperature. Blank disks were placed into a vial with 500 µL of a suspension of the compounds at 25 mg/mL and were left to soak for 30 min with inversion mixing every ∼10 min. The disks were then transferred to the bacterial culture plates (two replicates per compound per bacterial strain per each of two independent biological trials, for 4 replicates total). Plates were placed in 37°C incubator for 24 h, after which the zone of growth inhibition was measured. To address plate variation, the zone of inhibition measurement of the compounds was normalized to that of a disk containing zinc sulphate (disk soaked in 25 mg/mL solution).

Cell viability exposure assay was performed in liquid cultures. Defined dry weights of each of the compounds were added to either microbial culture tubes or 50 mL Erlenmeyer style culture flasks. Weights were determined to add equivalent molar masses of zinc to each culture, *i.e.*, the mole fraction of the zinc in ZnSO_4_^.^H_2_O was used to calculate the weight of the MOFs in order to have the same zinc load for each. For the pure aminocinnamic acids, the mole fraction mass weight of each aminocinnamic was calculated from the equivalent Zn-MOF, thus providing an equivalent organic load. This provides the ability to compare between the compounds in that if the MOFs dissociates completely, one could expect the same antimicrobial activity as the individual compound. LB media was added that then received a 1% inoculant from an overnight culture of the specific strain being tested. The cultures were incubated at 37°C for 18 h shaking at 150 rpm. Only the zinc sulphate appeared to fully dissolve in this aqueous environment, with MOFs being a suspension at least for the first hour of the experiment. Surviving viable cells were determined by log_10_ serial dilutions of the cultures which was then spot platted to LB agar plate that was incubated at 37°C overnight to determine the last dilution that provided growth. The data was then transformed to log_10_ killing compared to the unchallenged control. Experiments were performed in duplicate with two biological replicates at each of 0.5 and 2 mg/mL equivalents of zinc in 1 mL, 2 mL cultures.

Biofilm inhibition was evaluated from the cell viability cultures. Many bacteria will form a biofilm on the walls of culture tubes. The biomass of the biofilm (live cells + dead cells + plus matrix polymers) can be easily evaluated by staining with crystal violet (CV) (Allkja et al., 2020). The completed cultures from the cell viability exposure assay described above was decanted from the test tubes, which were rinsed 3 times with MilliQ quality water at double the culture volume. A 0.1% CV solution was added to each tube and left at room temperature for 30 min. The CV was decanted, and the test tubes rinsed 3 times with MilliQ quality water and inverted to allow to dry. Two mL of 30% acetic acid was then added to extract the bound CV. The amount of staining was then quantitated at an absorbance of 575 nm. Data is reported as % biofilm mass referenced to unchallenged culture.

## 3 Results and discussion

### 3.1 Synthesis and structural description

**FIGURE 1:**
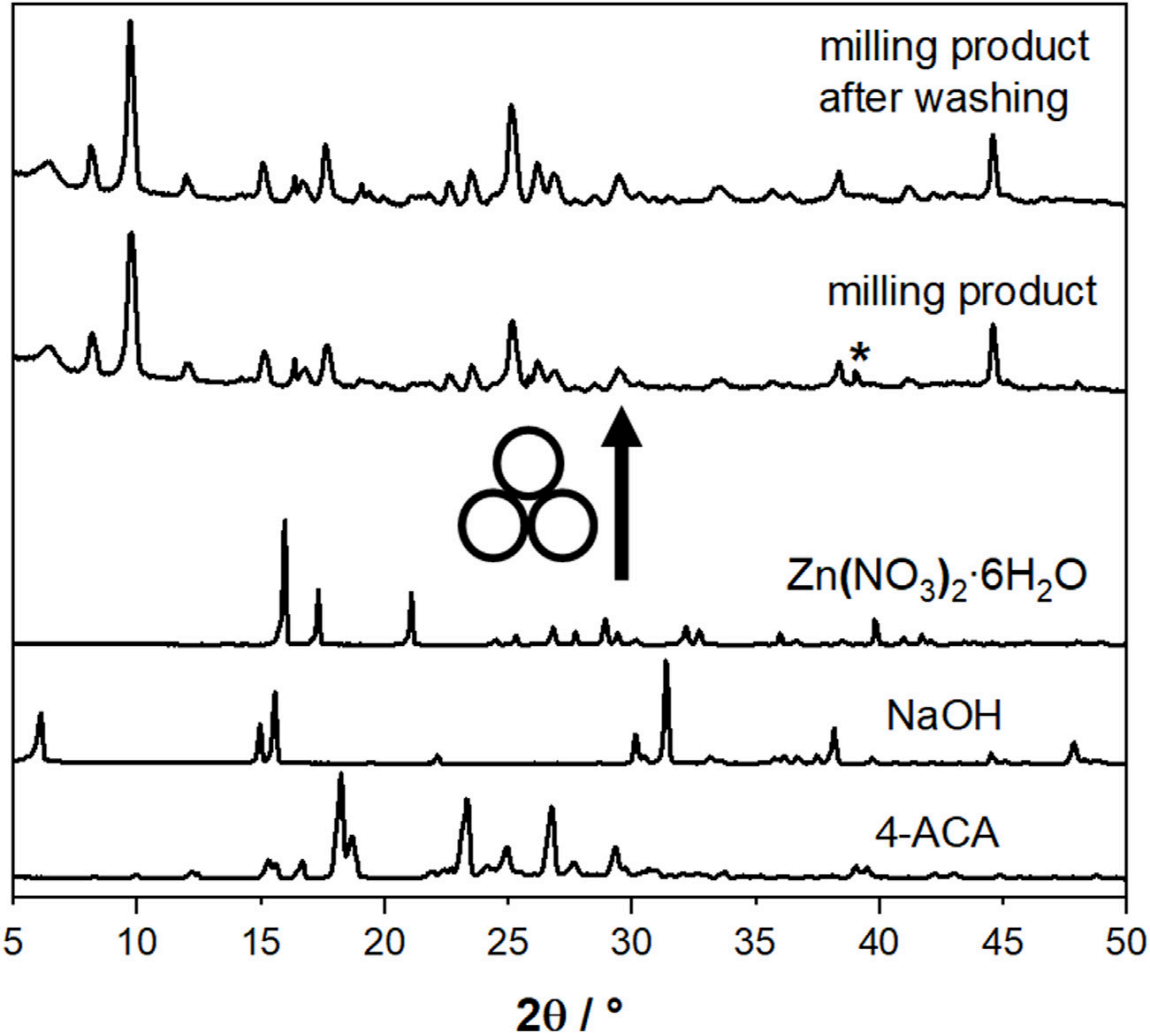
Comparison between the experimental PXRD patterns for the starting materials 4-ACA, NaOH, and Zn(NO_3_)_2_∙6H_2_O and for the milling product [Zn(4-AC)_2_]∙H_2_O] as prepared and after washing with water. The asterisk indicates the presence of NaNO_3_ as a by-product.

The synthetic method employed to generate the compounds presented in this study is characterized by its simplicity, cleanliness, and efficiency, involving few pivotal steps. Firstly, we explored the possibility of obtaining novel compounds through the mechanochemical reaction of Zn(NO_3_)_2_⋅6H_2_O, 3-cinnamic acid (3-ACA) or 4-cinnamic acid (4-ACA) and NaOH, in a 1:2:2 stoichiometric ratio (see Materials and methods). Figure 1 shows, as an example, a comparison between the experimental PXRD patterns for the starting materials 4-ACA, NaOH, and Zn(NO_3_)_2_·6H_2_O and for the milling product (later identified as [Zn(4-AC)_2_]·H_2_O]) as prepared and after washing with water to remove NaNO_3_.

The mechanochemical synthesis of possible analogues, starting either from 3-ACA and from an equimolar mixture of 3-ACA/4-ACA, was also explored. This approach also produced novel polycrystalline samples, that were characterized via PXRD (see Supplementary Figure S4). To fully characterize all the solid compounds obtained via mechanochemistry, we tried to grow single crystals by liquid diffusion. A neutral or slightly basic solution (pH = 7–8) of 3- or 4-cinnamate was carefully layered atop a zinc nitrate solution, with a layer of pure water placed in between to minimize interdiffusion and provide optimal conditions for crystal growth. Single crystals of good quality were thus obtained from the reactions involving 3-AC^-^ or 4-AC^-^, which were identified as [Zn(3-AC)_2_]⋅2H_2_O, [Zn(4-AC)_2_]⋅H_2_O, a 2D and a 3D MOFs, respectively. The powder diffraction patterns for all compounds, calculated on the basis of the single crystal structures, match the experimental ones, confirming that the same products are obtained *via* crystallization and mechanochemical reactions (see Supplementary Figures S3, S4).

When the liquid diffusion procedure was employed starting from an equimolar mixture of 3-AC^-^ and 4-AC^-^, however, no suitable single crystals could be obtained. A slight modification of the solution pH (from 7-8 to 6–6.5) yielded a different crystalline phase, later identified as [Zn(4-AC)_2_(H_2_O)_2_], with respect to the mechanochemical product. It is worth pointing out that although mechanochemical methods offer the advantage of eliminating or reducing the use of solvents (Braga et al., 2013; Friščić et al., 2020), the powders obtained *via* grinding/LAG suffered from poor crystallinity, as frequently observed for mechanochemically synthesized materials, due to the presence of nanostructures and defects generated during the process. Additionally, the syntheses were not fully reproducible, as in some instances incomplete reactivity was observed, or amorphous materials were recovered. The liquid diffusion approach was thus upscaled, with the goal of obtaining, for each solid phase, high quality and pure polycrystalline samples. TGA measurements with weight losses in the range of 4%–8% (Supplementary Figure S6) confirmed the presence of crystallization water as determined from XRD structural analysis in all cases (see below); for the product between Zn^2+^ and the 1:1 mixture 3-AC^-^/4-AC^-^ a weight loss of 0% indicated the presence of an anhydrous compound.

The structure of this compound was determined using powder XRD data, complemented by information from FTIR measurements. The FTIR spectra showed a shift from 1680 cm⁻^1^ (carboxylic acid) to 1635 cm⁻^1^ (carboxylate), consistent with other compounds in the series (Supplementary Figure S7). The structural analysis revealed the compound to be a 1D coordination polymer of formula [Zn(3-AC)(4-AC)].

The relevant structural features of all the synthesized complexes will be described below, starting with the 2D and 3D MOFs.

**FIGURE 2:**
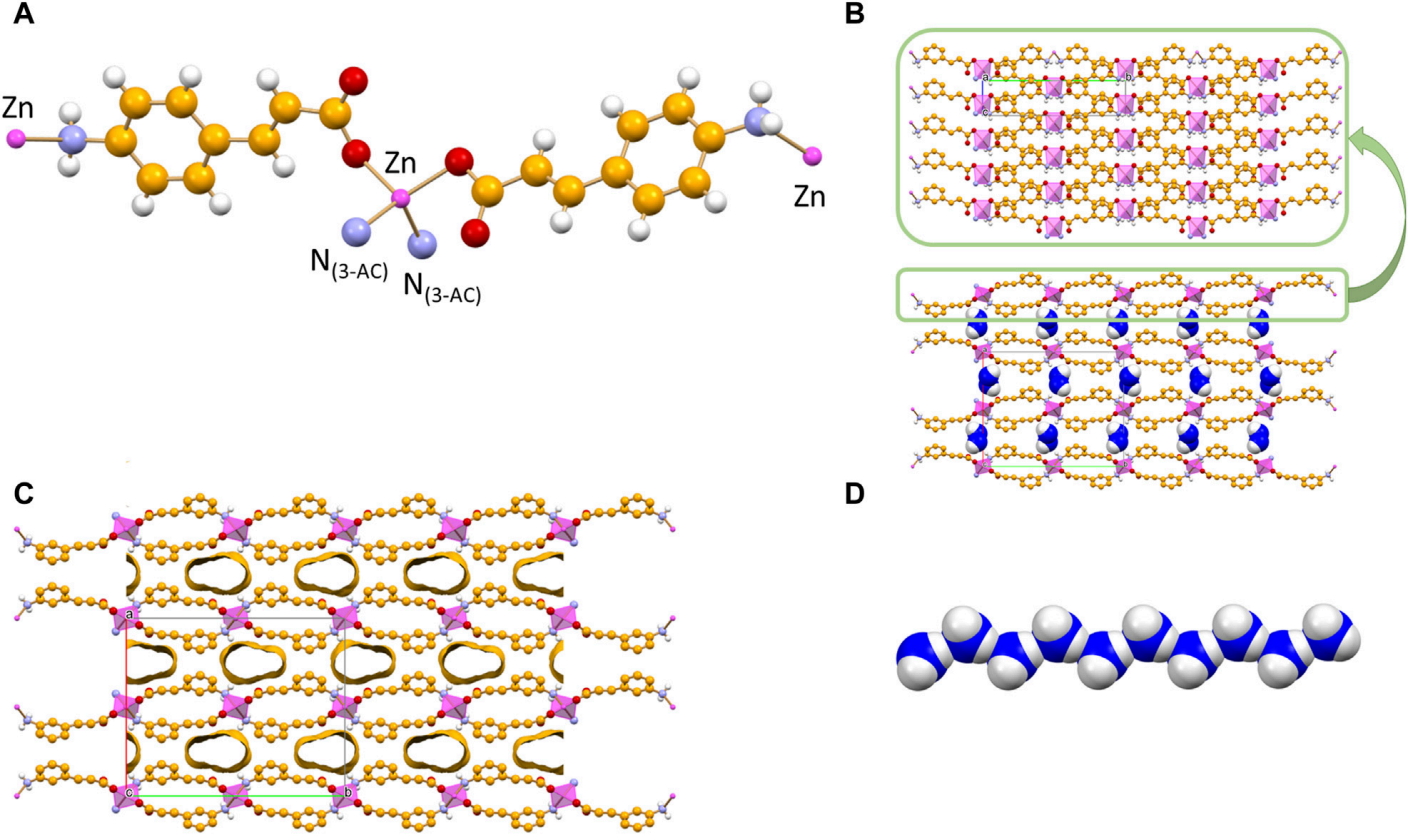
Tetrahedral coordination geometry of the 3-cinnamate anions around the zinc(II) cation in crystalline [Zn(3-AC)_2_]∙2H_2_O **(A)**. Stacking of 2D-layers **(B)** and a representation of the infinite channels **(C)**, each filled with a water molecules chain **(D)**. Coordination polyhedra around the zinc cation in purple, C atoms in orange, O_water_ atoms in blue. H_CH_ atoms omitted for clarity.

The 2D coordination polymer, two-dimensional MOF [Zn(3-AC)_2_]·2H_2_O crystallizes in the orthorhombic space group Aba2, with the zinc(II) cation located on a crystallographic two-fold axis; its main structural features are reported in Figure 2A. The zinc cation is tetrahedrally coordinated by two amino and two carboxylate groups belonging to four different 3-cinnamate anions. The overall structure consists of 2D sheets extending in the *bc*-plane (Figure 2B, top) and stacking along the *a*-axis (Figure 2B, bottom). The coordination polymer can be regarded as a 2D MOF, as the water molecules are arranged in channels along the *a*-axis direction, therefore the structure is that of a potentially porous material; the channels structure is evidenced in Figure 2C. Interestingly, the water molecules inside each channel form infinite hydrogen bonded chains (Figure 2D), with an O(H)···O distance of 2.862(7) Å, *i.e.*, analogous to the value found in solid water. The water molecules chain interacts on its side with the N- and O-atoms from the 3-AC^-^ ligand [O_w_···N_3-AC_- = 3.025(9)Å; O_w_···O_3-AC_- = 2.769(9) Å]; being “anchored” to the channels walls via hydrogen bonds, the water molecules spacing is perfectly periodical, and no positional disorder is observed along the chain.

**FIGURE 3:**
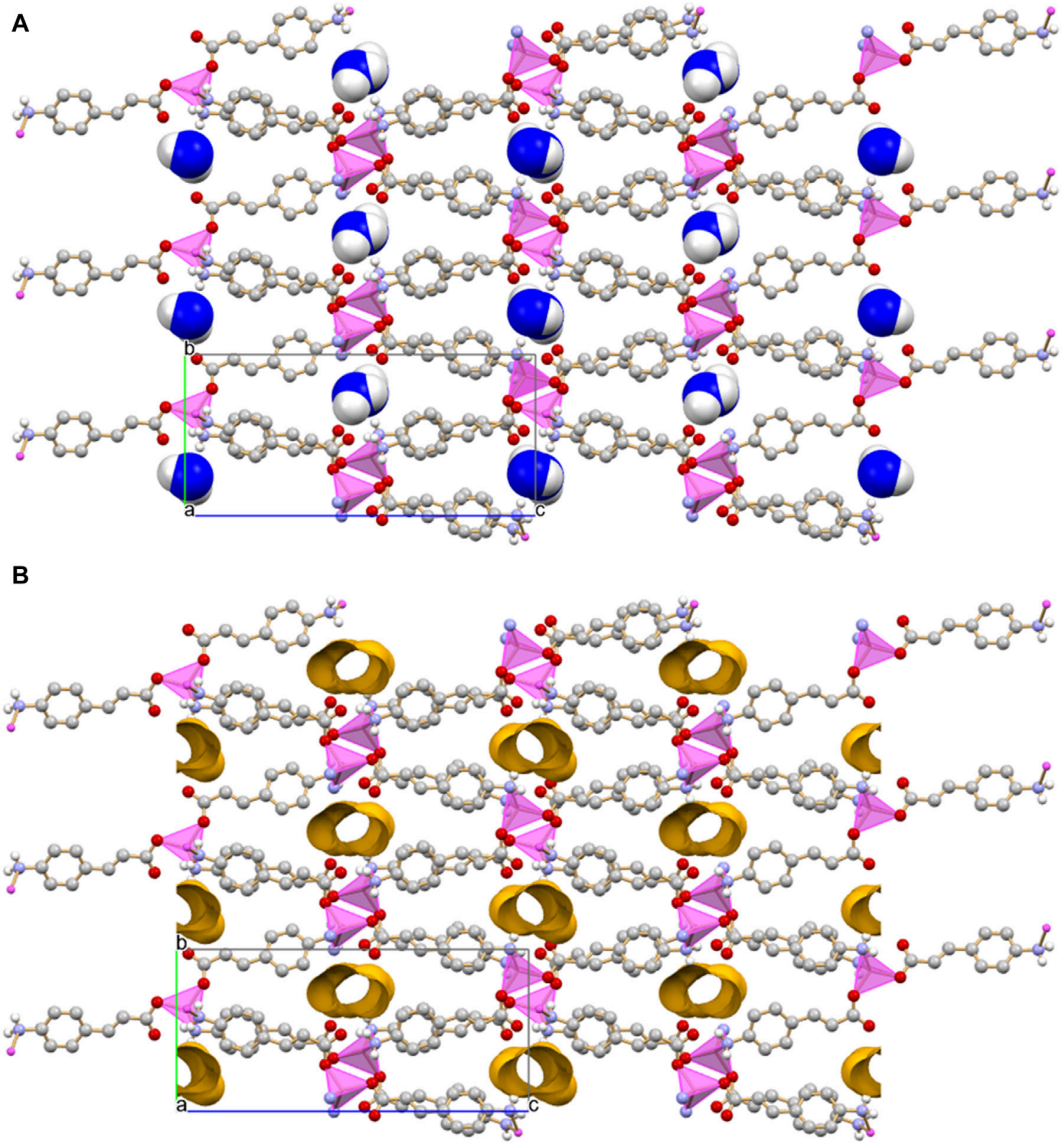
Infinite channels, shown here either filled with water molecules **(A)** and empty **(B)**, extend parallel to the *a*-axis direction in crystalline [Zn(4-AC)_2_]∙H_2_O. The water molecules inside the channel are largely spaced and they are at hydrogen bonding distance with the surrounding carboxylate and amino groups. Coordination polyhedra around the zinc cation in purple, empty channels evidenced in ochra, O_water_ atoms in blue. H_CH_ atoms omitted for clarity.

The monohydrated complex [Zn(4-AC)_2_]·H_2_O possesses the same stoichiometry of the complex with the 3-cynnamate anion, and the usual tetrahedral arrangement of ligands around the central zinc cation. However, the change in the relative position of the -NH_2_ group with respect to the carboxylate group determines a significant change in the packing features, with formation of a three-dimensional network that can be described as a 3D MOF: the crystal (Figure 3A) is characterized by the presence of infinite channels (Figure 3B), extending parallel to the crystallographic *a*-axis, containing only water molecules. Differently from what observed in [Zn(3-AC)_2_]·H_2_O, the water molecules are “loosely” arranged inside the channels, as can be appreciated from the long distance of almost 4 Å separating the oxygen atoms. The water molecules interact *via* hydrogen bonds with the surrounding carboxylate and amino groups belonging to the 4-cynnamate anions [O_w_···O_4-AC_- = 2.808(4), 2.988(5) Å; O_w_···N_3-AC_- = 2.959(6)Å].

**FIGURE 4:**
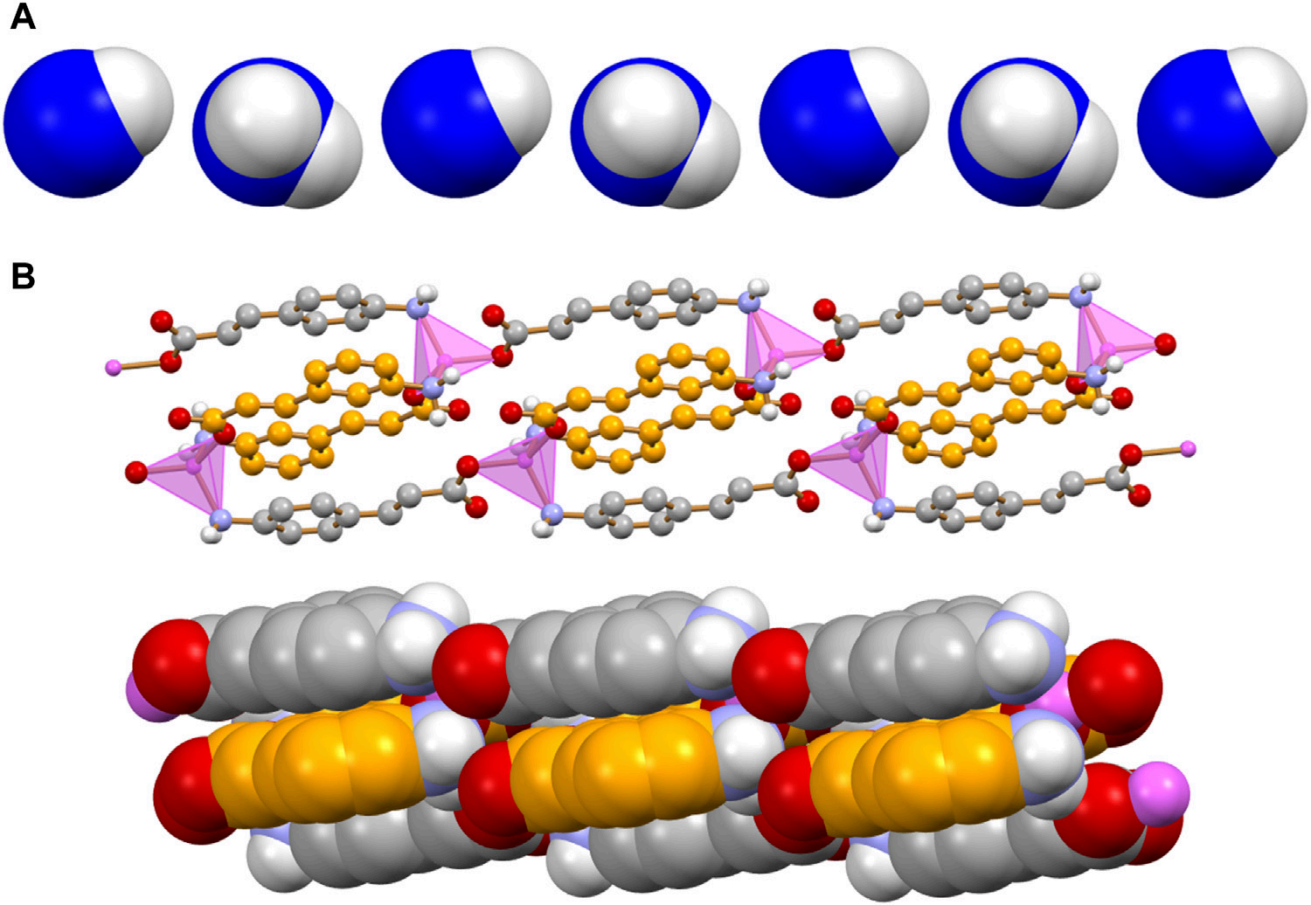
Main packing motif in crystalline [Zn(3-AC)(4-AC)]. Each 3- and 4-cinnamate ligand is bridging two Zn^2+^ cations; the resulting motif is an infinite ribbon **(A)** in which pairs of different cinnamate anions are at π-π stacking distance **(B)**. C atoms in orange and grey for the 3- and 4-cinnamate anions, respectively; zinc(II) in purple; H_CH_ atoms omitted for clarity.

The reaction of zinc(II) with an equimolar mixture of 3- and 4-cinnamate anions results in the formation of anhydrous [Zn(3-AC)(4-AC)], a 1D coordination polymer. The coordination geometry around the Zn^2+^ cation is that of a slightly distorted tetrahedron. Two divergent 3-AC^-^ anions (C-atoms in orange in Figure 4A) link two zinc cations in a sort of dimeric units, which are then linked via the 4-AC^-^ anions (C-atoms in grey in Figure 4A), resulting in a 1D coordination polymer. The aromatic moieties of the 3- and 4-cinnamate anions are at π-π stacking separation, as evidenced in Figure 4B.

**FIGURE 5:**
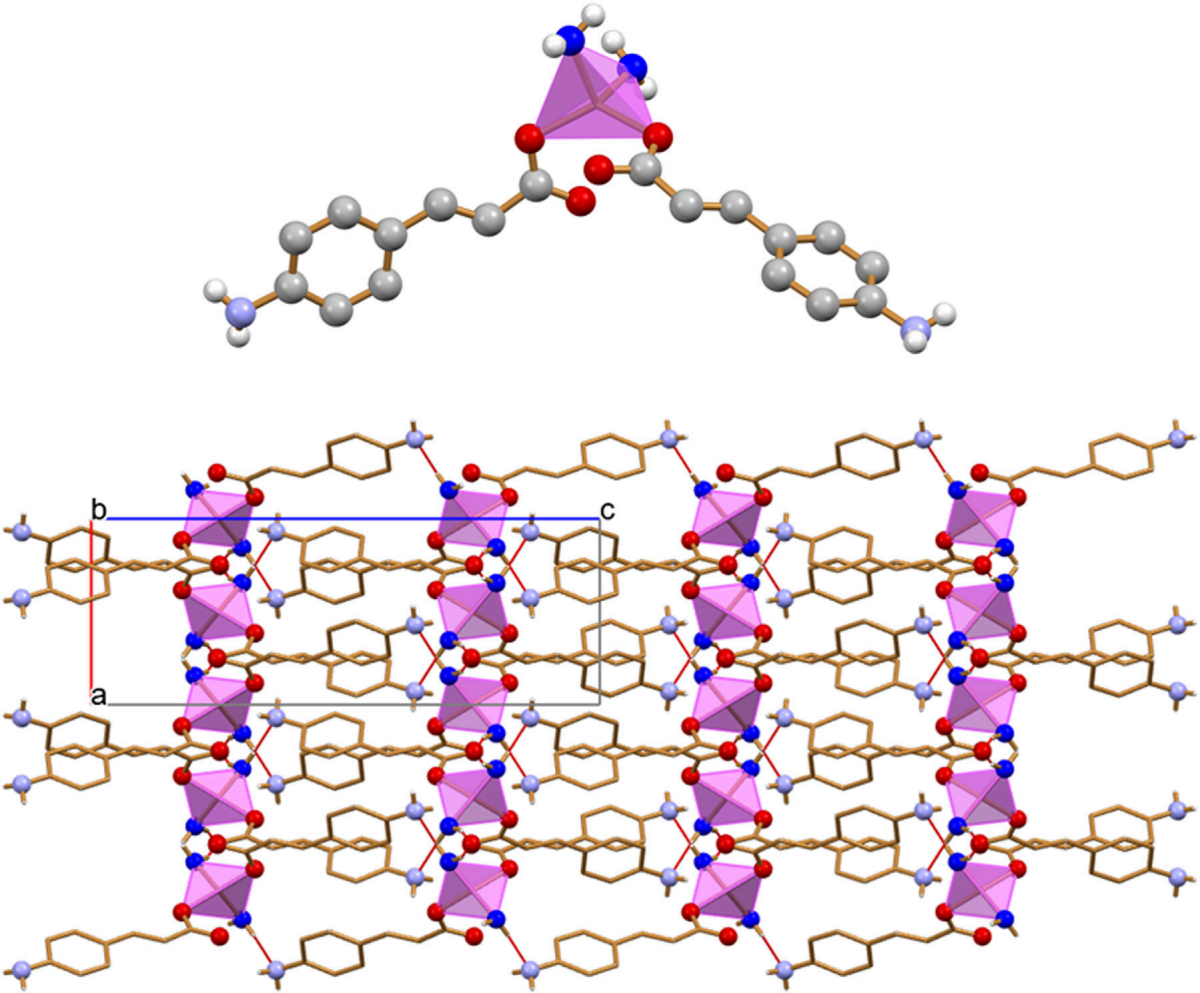
The 0D complex [Zn(4-AC)_2_(H_2_O)_2_] (top), and the 3D hydrogen bonding network (bottom) involving the water molecules bound to zinc and the carboxylate and amino groups belonging to adjacent 4-cynnamate ligands. [Zinc(II) in purple, O_water_ atoms in blue. H_CH_ atoms omitted for clarity].

The dimensionality of the zinc complex is reduced to 0D in crystalline [Zn(4-AC)_2_(H_2_O)_2_]: the addition of a stoichiometric water molecule, with respect to the 3D MOF [Zn(4-AC)_2_]⋅H_2_O], results in a completely different ligands arrangement (see Figure 5, top). The complex crystallizes in the orthorhombic Pbcn space group, with the zinc cation still in tetrahedral coordination, but in this case the coordination sphere is filled with two 4-cynnamate anions, bound to zinc *via* the carboxylate groups, and two water molecules [Zn^2+^···O_4-Ac_- = 1.932(2)Å; Zn^2+^···O_w_ = 1.985(2)Å]. Each water molecule interacts in turn *via* hydrogen bonds with two 4-AC^-^ anions belonging to neighboring complexes [O_w_··O_4-AC_- = 2.625(2)Å; O_w_··N_4-AC_- = 2.838(3)Å], thus giving rise to a 3D-network of hydrogen bonds (in red in Figure 5, bottom).

**SCHEME 2:**
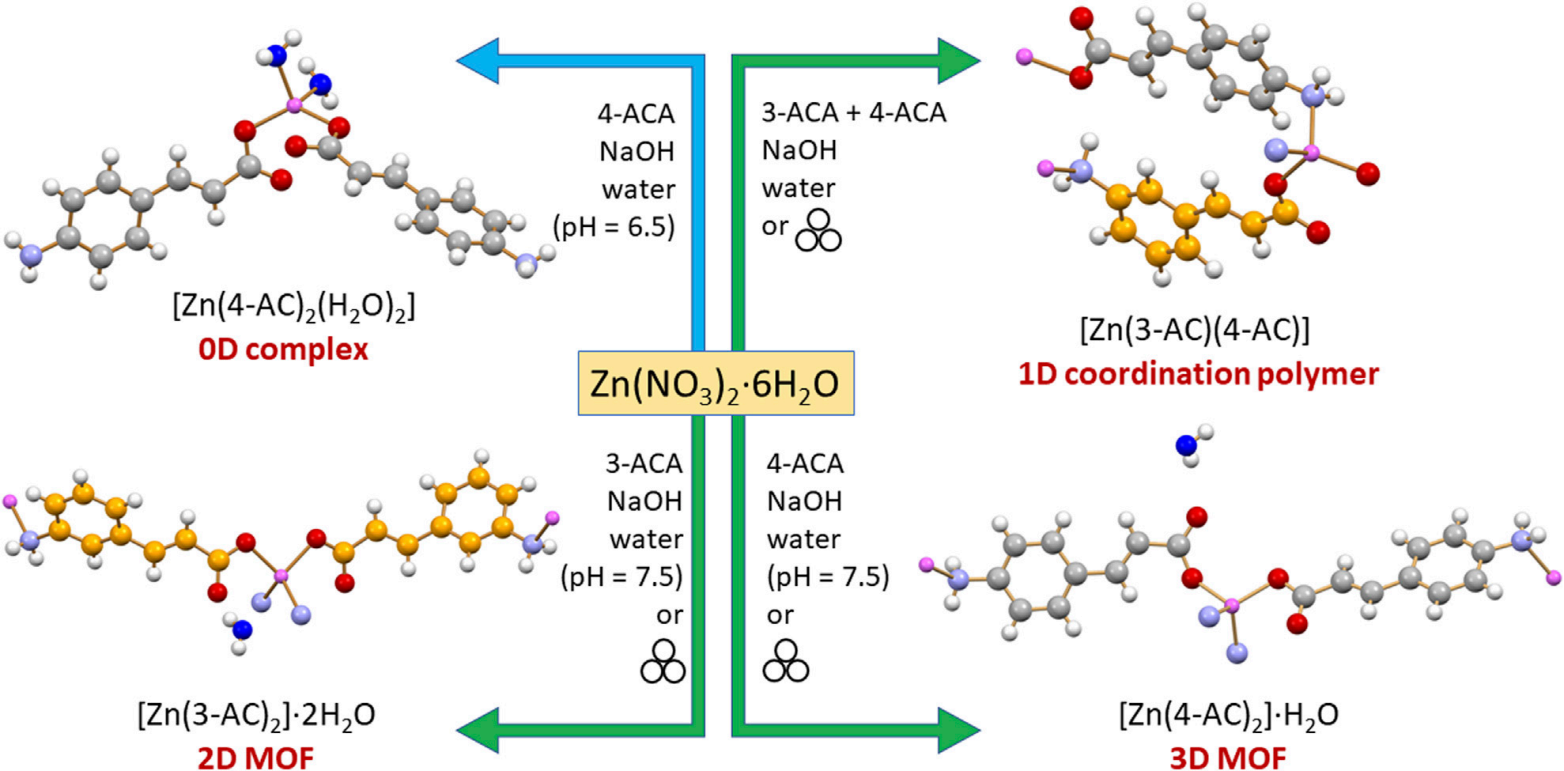
The solid-state products of mechanochemical and solution reactions of 4-ACA and 3-ACA with zinc nitrate.

Scheme 2 lists the solid compounds we have obtained via reaction of 4-aminocinnamate and 3-aminocinnamate ions with zinc(II) salts, i.e., [Zn(4-AC)_2_(H_2_O)_2_], a 0D complex, [Zn(3-AC)(4-AC)], a 1D coordination polymer, and the [Zn(3-AC)_2_]·2H_2_O and [Zn(4-AC)_2_]·H_2_O], which can be described as a 2D and a 3D MOF, respectively. All products were further tested for their antimicrobial efficacy against WHO bacteria of priority concern, *P. aeruginosa, E. coli, and S. aureus*.

### 3.2 Antimicrobial activity

As an initial evaluation to determine if any of the compounds or MOFs had antimicrobial activity a traditional Kirby-Bauer Disk diffusion assay was employed. This approach demonstrated antimicrobial activity of the MOFs against the three pathogen references strains, *P. aeruginosa, E. coli and S. aureus* (Supplementary Figure S8). However, the nature of this experiment expects that the compounds will adsorb to the filter disk then release from the disk into the hydrogel agar of the media plates and subsequently diffuse freely and that this would be the same for all compounds. Working with these compounds reflected that this is unlikely and as such this assay could not be considered quantitative nor comparative. Therefore, we explored other methods.

A traditional serial log_2_ dilution assay in a microtiter plate was also found to be impractical as reproducible pipetting of the compound suspensions was not possible (compounds adhering to the surface of the pipette tip), thus not allowing for reproductible minimum inhibitory concentration (MIC) determinations. Therefore, a cell viability assay was explored. In this assay as similar to traditional MIC, cultures were challenged at time zero of inoculation and surviving cells are then evaluated at a fixed later time. However, rather than a defined volume of a solution at a known concentration added to the culture. Dry material was weighed out using a precision analytical balance and added to the culture tube followed by media and inoculant. In most cases the compounds did not dissolve by the end of the experiment except for the zinc sulphate, with the MOFs in suspension. Experiments were performed at two different zinc equivalent concentrations (0.5 and 2 mg/mL). These concentrations were chosen based on the zinc MIC range of these strains, 1–16 mM ZnSO_4_^.^7H_2_O, cultured in various media conditions from previous work in our laboratory (Pormohammad and Turner, 2020).

The planktonic cell survivability at the high end of the zinc MIC exposure (2 mg/mL zinc) reflects very good log killing for all compounds (Supplementary Figure S9A). Good antimicrobial efficacy is considered if there is at least 3-fold log_10_ killing. We see the amount of killing appears the highest against *P. aeruginosa*, however, this strain grew to the highest cell density 10^12^ as it has robust growth. Thus, complete inhibition is seen with a 12 log_10_ killing. The density of the *E. coli* grew to 10^7^ and as such, this high zinc load gave complete cell growth inhibition from all compounds.

At the high concentration of antimicrobial we see that although there could be complete inhibition of planktonic growth, there can still be biofilm growth. The biofilm state of growth is well known for its high antimicrobial tolerance, even to antimicrobial metal ion challenge (Harrison et al., 2007). So, in the case of ZnSO_4_ exposure, although there was complete 12 log_10_ killing there was still ∼20% biofilm biomass for *P. aeruginosa,* and as much as 80% for *S. aureus* and *E. coli* (Supplementary Figure S9B) as compared to unexposed cultures. From this data we see that the cinnamic compounds on their own could not inhibit biofilm form of growth, but in the complex and MOF form they showed biofilm inhibition. This observation must be beyond the zinc activity alone, at least for the results observed for *S. aureus and E. coli.*

**FIGURE 6:**
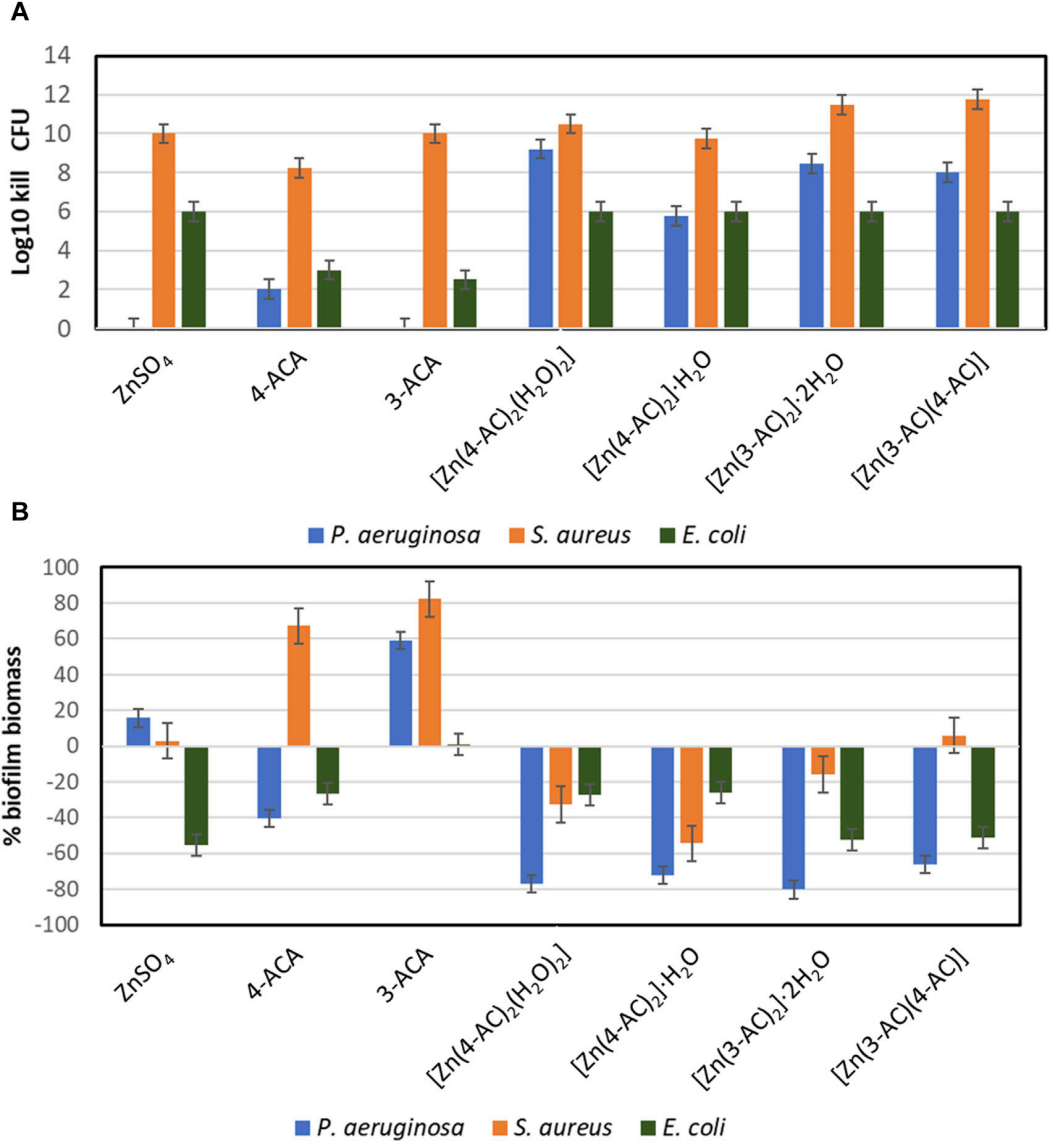
The Antimicrobial and antibiofilm activity of the compound. Concentration exposure at 0.5 mg/mL Zn equivalent levels. **(A)** Planktonic cell kill reflecting the culture viable cell density reduction in log_10_. **(B)** Evaluation of total biofilm density of cultures compared to unchallenged (averages and standard deviations from 4 replicates).

In order to capture subtle differences between the compounds, a concentration at the lower end of the MIC was chosen, 0.5 mg/mL zinc (Figure 6). It can be observed that at the lower exposure concentration there was no killing of planktonic *P. aeruginosa by* ZnSO_4_ (Figure 6A). Yet when zinc is in complex and MOFs with cinnamate ligands there is strong antimicrobial activity against all three bacterial strains.

With the 0.5 mg/mL zinc study we see an increase in biofilm biomass relative to unexposed for ZnSO_4_ as well as both 3-ACA and 4-ACA treatments. (Figure 6B). In this regard there is a slight increase in biofilm for *P. aeruginosa* yet no planktonic killing. This phenomenon has been observed for several antibiotics, where sublethal concentrations leads to increased biofilm (Kaplan, 2011; Andersson and Hughes, 2014). We also see this phenomenon highlighted in the ACA compounds without Zn as well, with the different compounds reflecting a differential effect to the three bacterial strains. Regardless the zinc-ACA compounds did not induce this effect and mostly reflected biofilm reduction (Figure 6B).

At this time, it is difficult to postulate a mechanism of the MOF. An initial impression is that there would be dissociation of the MOF into Zn^2+^ ions and the aminocinnamate ligands; in this case the degree of antimicrobial activity would be proportional to the dissociation rate. If this was true, the maximum antimicrobial activity observed would be equal to that of zinc sulphate alone. This appears to be the case for the log_10_ kill activity against *E. coli*. However, the efficacy is enhanced for two of the compounds against *S. aureus* and most mixtures showed enhanced activities against *P. aeruginosa*. Given the poor antimicrobial activity against *P. aeruginosa* of the aminocinnamic acids on their own, there must be something more going on beyond just additive effects of the individual constituents. This is even more dramatic when one considers the anti-biofilm activity. Our working hypothesis is that the MOF interacts with the surface of the bacteria, where, due to the physiology of the cell, the local physicochemical environment is different from the bulk media, thus leading to the dissociation of the MOF to smaller fragments or component molecules, followed by localized burst towards a high concentration of antimicrobials localized to the region around the cell.

## 4 Conclusion

In this work we have reported the synthesis, solid-state characterization, and antimicrobial evaluation of a novel series of coordination compounds. These compounds involve the combination of the mild antimicrobials 3- and 4-aminocynnamic acids with the zinc(II) metal ion.

Through solid-state and solution methods we successfully prepared four novel compounds: the 0D complex [Zn(4-AC)_2_(H_2_O)_2_], the 1D coordination polymer [Zn(3-AC)(4-AC)], the 2D MOF [Zn(3-AC)_2_]·2H_2_O, and the 3D MOF [Zn(4-AC)_2_]·H_2_O. Structural analyses, conducted by means of single crystal and powder X-ray diffraction, evidence the bidentate, divergent mode of complexation of 3- and 4-aminocynnamate anions, that can act as bridging ligands between different metal centers; in the 0D complex, featuring terminal ligands on the metal ion, the free function is involved in extended H-bonding networks throughout the crystal. Antimicrobial activity for all compounds was also tested against the reference strains of the pathogenic bacteria *P. aeruginosa*, *S. aureus*, and *E. coli*.

In summary, the synthesized Zn-compounds demonstrated equal or superior antimicrobial activity compared to the parent compounds against the tested reference strains of pathogenic bacteria. Unexpectedly, they also exhibited anti-biofilm activity. These results align with the objective stated in the introduction: finding new antimicrobial agents to address the increasing issue of antimicrobial resistance.

We posit that integrating the antimicrobial attributes of organic molecules with those of metal ions in coordination compounds may signify advancements in this endeavor. Nevertheless, these initial strides underscore the need for thorough contemplation to comprehend the mechanisms through which co-crystals and coordination polymers act against bacteria. Ongoing studies demanding additional specialized and collaborative research are currently underway to delve deeper into these aspects.

## Data Availability

The datasets presented in this study can be found in online repositories. The names of the repository/repositories and accession number(s) can be found below: https://www.ccdc.cam.ac.uk/solutions/csd-system/components/csd/, 2350358-2350363.
