# Supplementary material for: From 0D-complex to 3D-MOF: changing the antimicrobial activity of zinc(II) via reaction with aminocinnamic acids
Source: Front Chem. 2024 Jul 8;12:1430457. doi: 10.3389/fchem.2024.1430457 (PMC11260639; doi:10.3389/fchem.2024.1430457)

checkCIF/PLATON report

Structure factors have been supplied for datablock(s) 2-neutral, 2-zwitterion, Zn-0D, Zn-2D, Zn-3D

THIS REPORT IS FOR GUIDANCE ONLY. IF USED AS PART OF A REVIEW PROCEDURE FOR PUBLICATION, IT SHOULD NOT REPLACE THE EXPERTISE OF AN EXPERIENCED CRYSTALLOGRAPHIC REFEREE.

No syntax errors found.   CIF dictionary   Interpreting this report

Datablock: 2-neutral

Bond precision: C-C = 0.0053 A   Wavelength=0.71073

Cell:	a=14.4183(17)	b=5.0308(6)	c=23.208(3)
	alpha=90	beta=102.107(11)	gamma=90
Temperature:	300 K		

	Calculated	Reported
Volume	1646.0(4)	1645.9(3)
Space group	P 21/n	P 1 21/n 1
Hall group	-P 2yn	-P 2yn
Moiety formula	C9 H9 N O2	C9 H9 N O2
Sum formula	C9 H9 N O2	C9 H9 N O2
Mr	163.17	163.17
Dx,g cm-3	1.317	1.317
Z	8	8
Mu (mm-1)	0.094	0.094
F000	688.0	688.0
F000'	688.34	
h,k,lmax	19,6,32	19,6,30
Nref	4538	3766
Tmin,Tmax	0.994,0.997	0.986,1.000
Tmin'	0.993	

Correction method= # Reported T Limits: Tmin=0.986 Tmax=1.000
AbsCorr = MULTI-SCAN

Data completeness= 0.830   Theta(max)= 29.446

R(reflections)= 0.0757(1520)   wR2(reflections)= 0.1893(3766)

S = 0.938   Npar= 238

The following ALERTS were generated. Each ALERT has the format
test-name_ALERT_alert-type_alert-level.
Click on the hyperlinks for more details of the test.

Alert level C

```
PLAT026_ALERT_3_C Ratio Observed / Unique Reflections (too) Low ..        40% Check
PLAT340_ALERT_3_C Low Bond Precision on  C-C Bonds ...............    0.00528 Ang.
PLAT353_ALERT_3_C Long   N-H (N0.87,N1.01A)  N2       - H2A      .       1.01 Ang.
PLAT790_ALERT_4_C Centre of Gravity not Within Unit Cell: Resd.  #          1 Note
              C9 H9 N O2
PLAT906_ALERT_3_C Large K Value in the Analysis of Variance ......     29.200 Check
PLAT906_ALERT_3_C Large K Value in the Analysis of Variance ......      5.526 Check
PLAT906_ALERT_3_C Large K Value in the Analysis of Variance ......      2.110 Check
PLAT910_ALERT_3_C Missing # of FCF Reflection(s) Below Theta(Min).          6 Note
                2  0  0,  -1  0  1,   1  0  1,  -2  0  2,   0  0  2,  -1  0  3,
```

Alert level G

```
PLAT002_ALERT_2_G Number of Distance or Angle Restraints on AtSite          2 Note
PLAT007_ALERT_5_G Number of Unrefined Donor-H Atoms .............           1 Report
              H1
PLAT066_ALERT_1_G Predicted and Reported Tmin&Tmax Range Identical          ? Check
PLAT172_ALERT_4_G The CIF-Embedded .res File Contains DFIX Records          1 Report
PLAT790_ALERT_4_G Centre of Gravity not Within Unit Cell: Resd.  #          2 Note
              C9 H9 N O2
PLAT860_ALERT_3_G Number of Least-Squares Restraints ............           1 Note
PLAT912_ALERT_4_G Missing # of FCF Reflections Above STh/L=  0.600        751 Note
PLAT941_ALERT_3_G Average HKL Measurement Multiplicity ..........         1.8 Low
PLAT952_ALERT_5_G Calculated (ThMax) and CIF-Reported Lmax Differ.          2 Units
PLAT958_ALERT_1_G Calculated (ThMax) and Actual (FCF) Lmax Differ.          2 Units
PLAT969_ALERT_5_G The 'Henn et al.' R-Factor-gap value ..........        2.72 Note
              Predicted wR2: Based on SigI**2  6.97 or SHELX Weight 20.84
PLAT978_ALERT_2_G Number C-C Bonds with Positive Residual Density.          0 Info
```

0 **ALERT level A** = Most likely a serious problem - resolve or explain
0 **ALERT level B** = A potentially serious problem, consider carefully
8 **ALERT level C** = Check. Ensure it is not caused by an omission or oversight
12 **ALERT level G** = General information/check it is not something unexpected

2 ALERT type 1 CIF construction/syntax error, inconsistent or missing data
2 ALERT type 2 Indicator that the structure model may be wrong or deficient
9 ALERT type 3 Indicator that the structure quality may be low
4 ALERT type 4 Improvement, methodology, query or suggestion
3 ALERT type 5 Informative message, check

Datablock: 2-zwitterion

Bond precision: C-C = 0.0047 A   Wavelength=0.71073

Cell:	a=4.2692(6)	b=9.7343(13)	c=10.1777(14)
	alpha=111.122(13)	beta=90.504(11)	gamma=90.661(11)
Temperature:	293 K		

	Calculated	Reported
Volume	394.48(10)	394.48(10)
Space group	P -1	P -1
Hall group	-P 1	-P 1
Moiety formula	C9 H9 N O2	C9 H9 N O2
Sum formula	C9 H9 N O2	C9 H9 N O2
Mr	163.17	163.17
Dx,g cm-3	1.374	1.374
Z	2	2
Mu (mm-1)	0.098	0.098
F000	172.0	172.0
F000'	172.09	
h,k,lmax	5,13,14	5,13,13
Nref	2163	1826
Tmin,Tmax	0.994,0.997	0.184,1.000
Tmin'	0.992	

Correction method= # Reported T Limits: Tmin=0.184 Tmax=1.000
AbsCorr = MULTI-SCAN

Data completeness= 0.844 Theta(max)= 29.313

R(reflections)= 0.0769(963) wR2(reflections)= 0.2100(1826)

S = 1.067 Npar= 110

The following ALERTS were generated. Each ALERT has the format
test-name_ALERT_alert-type_alert-level.
Click on the hyperlinks for more details of the test.

Alert level C

```
PLAT250_ALERT_2_C Large U3/U1 Ratio for <U(i,j)> Tensor(Resd    1)        2.3 Note
PLAT340_ALERT_3_C Low Bond Precision on  C-C Bonds ...............    0.00467 Ang.
PLAT906_ALERT_3_C Large K Value in the Analysis of Variance ......     56.054 Check
PLAT906_ALERT_3_C Large K Value in the Analysis of Variance ......      6.537 Check
PLAT906_ALERT_3_C Large K Value in the Analysis of Variance ......      2.931 Check
```

Alert level G

```
PLAT007_ALERT_5_G Number of Unrefined Donor-H Atoms .............          3 Report
              H1A   H1B   H1C
PLAT199_ALERT_1_G Reported _cell_measurement_temperature ..... (K)        293 Check
```

```
PLAT200_ALERT_1_G Reported   _diffrn_ambient_temperature ..... (K)        293 Check
PLAT910_ALERT_3_G Missing # of FCF Reflection(s) Below Theta(Min).          3 Note
                0  1  0,   0 -1  1,   0  0  1,
PLAT912_ALERT_4_G Missing # of FCF Reflections Above STh/L=  0.600        332 Note
PLAT941_ALERT_3_G Average HKL Measurement Multiplicity ..........         1.6 Low
PLAT969_ALERT_5_G The 'Henn et al.' R-Factor-gap value ..........        2.46 Note
              Predicted wR2: Based on SigI**2  8.55 or SHELX Weight 20.31
PLAT978_ALERT_2_G Number C-C Bonds with Positive Residual Density.          1 Info
```

```
   0 ALERT level A = Most likely a serious problem - resolve or explain
   0 ALERT level B = A potentially serious problem, consider carefully
   5 ALERT level C = Check. Ensure it is not caused by an omission or oversight
   8 ALERT level G = General information/check it is not something unexpected

   2 ALERT type 1 CIF construction/syntax error, inconsistent or missing data
   2 ALERT type 2 Indicator that the structure model may be wrong or deficient
   6 ALERT type 3 Indicator that the structure quality may be low
   1 ALERT type 4 Improvement, methodology, query or suggestion
   2 ALERT type 5 Informative message, check
```

Datablock: Zn-0D

Bond precision: C-C = 0.0029 A   Wavelength=0.71073

Cell:	a=7.5978(2)	b=11.4111(3)	c=20.8074(6)
	alpha=90	beta=90	gamma=90
Temperature:	300 K		

	Calculated	Reported
Volume	1803.99(9)	1803.99(8)
Space group	P b c n	P b c n
Hall group	-P 2n 2ab	-P 2n 2ab
Moiety formula	C18 H20 N2 O6 Zn	C18 H20 N2 O6 Zn
Sum formula	C18 H20 N2 O6 Zn	C18 H20 N2 O6 Zn
Mr	425.75	425.73
Dx,g cm-3	1.568	1.567
Z	4	4
Mu (mm-1)	1.400	1.400
F000	880.0	880.0
F000'	881.52	
h,k,lmax	10,15,28	10,15,26
Nref	2498	2289
Tmin,Tmax	0.919,0.959	0.900,1.000
Tmin'	0.869	

Correction method= # Reported T Limits: Tmin=0.900 Tmax=1.000
AbsCorr = MULTI-SCAN

Data completeness= 0.916 Theta(max)= 29.418

R(reflections)= 0.0322(1656) wR2(reflections)= 0.0871(2289)

S = 0.968 Npar= 136

The following ALERTS were generated. Each ALERT has the format
test-name_ALERT_alert-type_alert-level.
Click on the hyperlinks for more details of the test.

Alert level C

```
PLAT420_ALERT_2_C D-H Bond Without Acceptor  N1       --H1A      .     Please Check
PLAT420_ALERT_2_C D-H Bond Without Acceptor  N1       --H6       .     Please Check
PLAT906_ALERT_3_C Large K Value in the Analysis of Variance ......      3.655 Check
```

Alert level G

```
PLAT003_ALERT_2_G Number of Uiso or Uij Restrained non-H Atoms ...         14 Report
PLAT007_ALERT_5_G Number of Unrefined Donor-H Atoms .............          1 Report
              H0AA
PLAT187_ALERT_4_G The CIF-Embedded .res File Contains RIGU Records          1 Report
PLAT720_ALERT_4_G Number of Unusual/Non-Standard Labels ..........         3 Note
              O0A     H0AA    H0AB
PLAT794_ALERT_5_G Tentative Bond Valency for Zn1       (II)      .       2.02 Info
PLAT860_ALERT_3_G Number of Least-Squares Restraints ............        93 Note
PLAT912_ALERT_4_G Missing # of FCF Reflections Above STh/L=  0.600       187 Note
PLAT952_ALERT_5_G Calculated (ThMax) and CIF-Reported Lmax Differ.         2 Units
PLAT958_ALERT_1_G Calculated (ThMax) and Actual (FCF) Lmax Differ.         2 Units
PLAT969_ALERT_5_G The 'Henn et al.' R-Factor-gap value ..........       3.72 Note
              Predicted wR2: Based on SigI**2  2.34 or SHELX Weight  9.27
PLAT978_ALERT_2_G Number C-C Bonds with Positive Residual Density.          6 Info
```

---

0 **ALERT level A** = Most likely a serious problem - resolve or explain
0 **ALERT level B** = A potentially serious problem, consider carefully
3 **ALERT level C** = Check. Ensure it is not caused by an omission or oversight
11 **ALERT level G** = General information/check it is not something unexpected

1 ALERT type 1 CIF construction/syntax error, inconsistent or missing data
4 ALERT type 2 Indicator that the structure model may be wrong or deficient
2 ALERT type 3 Indicator that the structure quality may be low
3 ALERT type 4 Improvement, methodology, query or suggestion
4 ALERT type 5 Informative message, check

Datablock: Zn-2D

Bond precision: C-C = 0.0093 A Wavelength=0.71073

Cell:	a=17.1162(14)	b=21.0447(11)	c=5.1540(3)
	alpha=90	beta=90	gamma=90
Temperature:	295 K		

	Calculated	Reported
Volume	1856.5(2)	1856.5(2)
Space group	A b a 2	A e a 2
Hall group	A 2 -2ac	A 2 -2ac
Moiety formula	C18 H16 N2 O4 Zn, 2(H2 O)	C18 H16 N2 O4 Zn, 2(H2 O)
Sum formula	C18 H20 N2 O6 Zn	C18 H20 N2 O6 Zn
Mr	425.75	425.73
Dx,g cm-3	1.523	1.523
Z	4	4
Mu (mm-1)	1.360	1.360
F000	880.0	880.0
F000'	881.52	
h,k,lmax	23,28,7	23,28,7
Nref	2462[1366]	2021
Tmin,Tmax	0.952,0.973	0.986,1.000
Tmin'	0.934	

Correction method= # Reported T Limits: Tmin=0.986 Tmax=1.000
AbsCorr = MULTI-SCAN

Data completeness= 1.48/0.82 Theta(max)= 28.994

R(reflections)= 0.0590(1273) wR2(reflections)= 0.0914(2021)

S = 1.007 Npar= 126

The following ALERTS were generated. Each ALERT has the format
test-name_ALERT_alert-type_alert-level.
Click on the hyperlinks for more details of the test.

Alert level C

```
PLAT341_ALERT_3_C Low Bond Precision on  C-C Bonds ..............    0.00933 Ang.
PLAT767_ALERT_4_C INS Embedded LIST 6 Instruction Should be LIST 4    Please Check
PLAT906_ALERT_3_C Large K Value in the Analysis of Variance ......     2.662 Check
PLAT910_ALERT_3_C Missing # of FCF Reflection(s) Below Theta(Min).         5 Note
                2  0  0,   0  2  0,   1  2  0,   2  2  0,   0  4  0,
PLAT911_ALERT_3_C Missing FCF Refl Between Thmin & STh/L=    0.600         6 Report
                9 20  0,  10 20  0,  11 20  0,  12 20  0,   9 22  0,  10 22  0,
```

Alert level G

```
PLAT004_ALERT_5_G Polymeric Structure Found with Maximum Dimension          2 Info
PLAT007_ALERT_5_G Number of Unrefined Donor-H Atoms .............          4 Report
              H1A   H1B   H3A   H3B
PLAT187_ALERT_4_G The CIF-Embedded .res File Contains RIGU Records          2 Report
PLAT794_ALERT_5_G Tentative Bond Valency for Zn1       (II)      .       1.76 Info
PLAT860_ALERT_3_G Number of Least-Squares Restraints ............         88 Note
PLAT912_ALERT_4_G Missing # of FCF Reflections Above STh/L=  0.600        152 Note
PLAT915_ALERT_3_G No Flack x Check Done: Low Friedel Pair Coverage         75 %
PLAT941_ALERT_3_G Average HKL Measurement Multiplicity ..........         3.1 Low
PLAT969_ALERT_5_G The 'Henn et al.' R-Factor-gap value ..........        1.39 Note
              Predicted wR2: Based on SigI**2  6.57 or SHELX Weight  9.37
PLAT978_ALERT_2_G Number C-C Bonds with Positive Residual Density.          0 Info
```

---

0 **ALERT level A** = Most likely a serious problem - resolve or explain
0 **ALERT level B** = A potentially serious problem, consider carefully
5 **ALERT level C** = Check. Ensure it is not caused by an omission or oversight
10 **ALERT level G** = General information/check it is not something unexpected

0 ALERT type 1 CIF construction/syntax error, inconsistent or missing data
1 ALERT type 2 Indicator that the structure model may be wrong or deficient
7 ALERT type 3 Indicator that the structure quality may be low
3 ALERT type 4 Improvement, methodology, query or suggestion
4 ALERT type 5 Informative message, check

Datablock: Zn-3D

Bond precision: C-C = 0.0091 A   Wavelength=0.71073

Cell:	a=7.7157(6)	b=10.1066(7)	c=21.9887(12)
	alpha=90	beta=90	gamma=90
Temperature:	298 K		

	Calculated	Reported
Volume	1714.7(2)	1714.7(2)
Space group	P 21 21 21	P 21 21 21
Hall group	P 2ac 2ab	P 2ac 2ab
Moiety formula	C18 H16 N2 O4 Zn, H2 O	C18 H16 N2 O4 Zn, H2 O
Sum formula	C18 H18 N2 O5 Zn	C18 H18 N2 O5 Zn
Mr	407.73	407.71
Dx,g cm-3	1.579	1.579
Z	4	4
Mu (mm-1)	1.465	1.465
F000	840.0	840.0
F000’	841.48	
h,k,lmax	10,14,30	10,13,28
Nref	4800[2741]	3366
Tmin,Tmax	0.932,0.957	0.945,1.000
Tmin’	0.929	

Correction method= # Reported T Limits: Tmin=0.945 Tmax=1.000
AbsCorr = MULTI-SCAN

Data completeness= 1.23/0.70 Theta(max)= 29.555

R(reflections)= 0.0572(2312) wR2(reflections)= 0.0911(3366)

S = 1.002 Npar= 227

The following ALERTS were generated. Each ALERT has the format
test-name_ALERT_alert-type_alert-level.
Click on the hyperlinks for more details of the test.

Alert level C

```
PLAT218_ALERT_3_C Constrained U(ij) Components(s) for O5         .          6 Check
PLAT241_ALERT_2_C High   'MainMol' Ueq as Compared to Neighbors of         O4 Check
PLAT260_ALERT_2_C Large Average Ueq of Residue Including        O5      0.182 Check
PLAT341_ALERT_3_C Low Bond Precision on  C-C Bonds ..............    0.00911 Ang.
PLAT906_ALERT_3_C Large K Value in the Analysis of Variance ......      2.580 Check
PLAT910_ALERT_3_C Missing # of FCF Reflection(s) Below Theta(Min).          5 Note
                1  0  1,   0  1  1,   0  0  2,   1  0  2,   0  1  2,
PLAT915_ALERT_3_C No Flack x Check Done: Low Friedel Pair Coverage         54 %
PLAT975_ALERT_2_C Check Calcd Resid. Dens.  0.84Ang From O5      .       0.41 eA-3
```

Alert level G

```
PLAT004_ALERT_5_G Polymeric Structure Found with Maximum Dimension          3 Info
PLAT007_ALERT_5_G Number of Unrefined Donor-H Atoms .............          6 Report
              H1A   H1B   H2A   H2B   H5A   H5B
PLAT033_ALERT_4_G Flack x Value Deviates > 3.0 * sigma from Zero .      0.190 Note
PLAT169_ALERT_4_G The CIF-Embedded .res File Contains AFIX 1 Recds          3 Report
```

```
PLAT187_ALERT_4_G The CIF-Embedded .res File Contains RIGU Records          1 Report
PLAT794_ALERT_5_G Tentative Bond Valency for Zn1       (II)      .       1.88 Info
PLAT912_ALERT_4_G Missing # of FCF Reflections Above STh/L=  0.600        449 Note
PLAT941_ALERT_3_G Average HKL Measurement Multiplicity ..........         2.2 Low
PLAT952_ALERT_5_G Calculated (ThMax) and CIF-Reported Lmax Differ.          2 Units
PLAT958_ALERT_1_G Calculated (ThMax) and Actual (FCF) Lmax Differ.          2 Units
PLAT969_ALERT_5_G The 'Henn et al.' R-Factor-gap value ..........        1.39 Note
              Predicted wR2: Based on SigI**2  6.57 or SHELX Weight  9.41
PLAT978_ALERT_2_G Number C-C Bonds with Positive Residual Density.          0 Info
```

```
   0 ALERT level A = Most likely a serious problem - resolve or explain
   0 ALERT level B = A potentially serious problem, consider carefully
   8 ALERT level C = Check. Ensure it is not caused by an omission or oversight
  12 ALERT level G = General information/check it is not something unexpected

   1 ALERT type 1 CIF construction/syntax error, inconsistent or missing data
   4 ALERT type 2 Indicator that the structure model may be wrong or deficient
   6 ALERT type 3 Indicator that the structure quality may be low
   4 ALERT type 4 Improvement, methodology, query or suggestion
   5 ALERT type 5 Informative message, check
```

It is advisable to attempt to resolve as many as possible of the alerts in all categories. Often the minor alerts point to easily fixed oversights, errors and omissions in your CIF or refinement strategy, so attention to these fine details can be worthwhile. In order to resolve some of the more serious problems it may be necessary to carry out additional measurements or structure refinements. However, the purpose of your study may justify the reported deviations and the more serious of these should normally be commented upon in the discussion or experimental section of a paper or in the "special_details" fields of the CIF. checkCIF was carefully designed to identify outliers and unusual parameters, but every test has its limitations and alerts that are not important in a particular case may appear. Conversely, the absence of alerts does not guarantee there are no aspects of the results needing attention. It is up to the individual to critically assess their own results and, if necessary, seek expert advice.

Publication of your CIF in IUCr journals

A basic structural check has been run on your CIF. These basic checks will be run on all CIFs submitted for publication in IUCr journals (*Acta Crystallographica*, *Journal of Applied Crystallography*, *Journal of Synchrotron Radiation*); however, if you intend to submit to *Acta Crystallographica Section C* or *E* or *IUCrData*, you should make sure that full publication checks are run on the final version of your CIF prior to submission.

Publication of your CIF in other journals

Please refer to the *Notes for Authors* of the relevant journal for any special instructions relating to CIF submission.

PLATON version of 06/01/2024; check.def file version of 05/01/2024

Datablock 2-neutral - ellipsoid plot

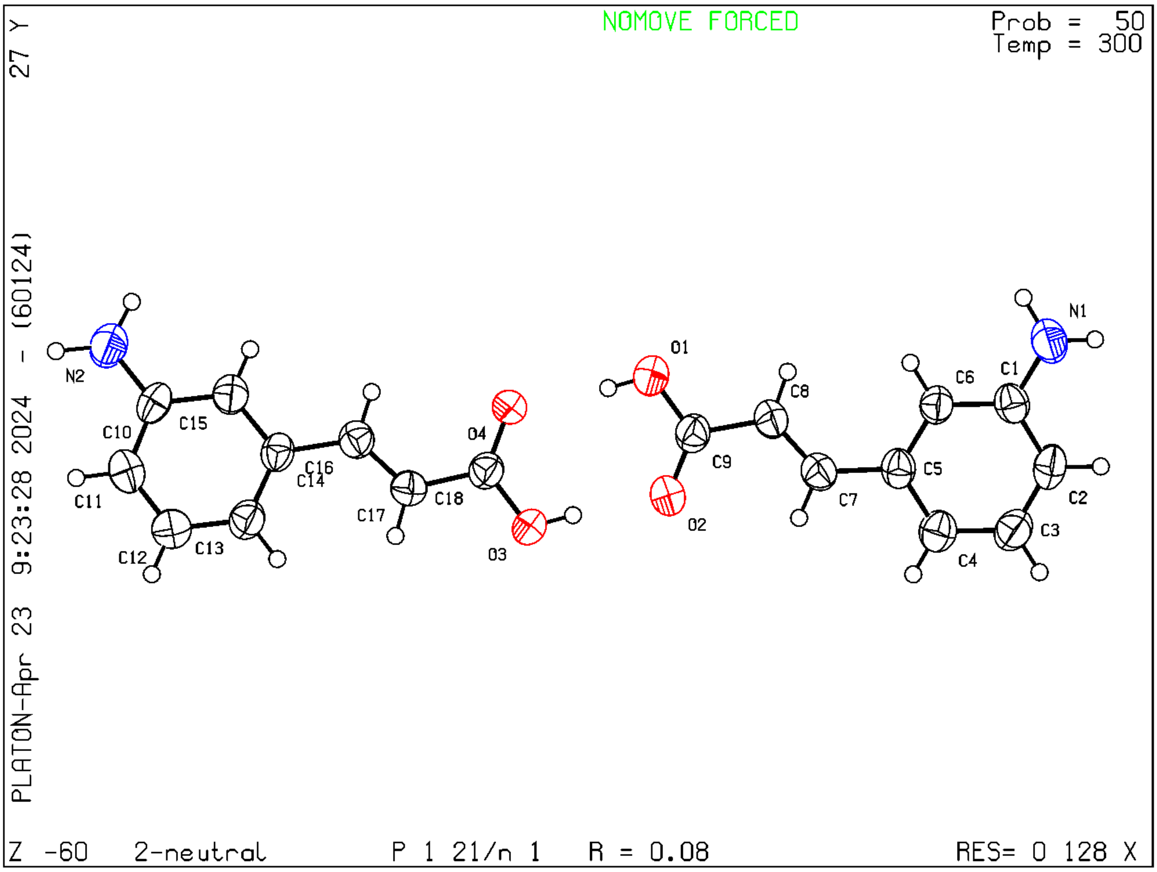

Datablock 2-zwitterion - ellipsoid plot

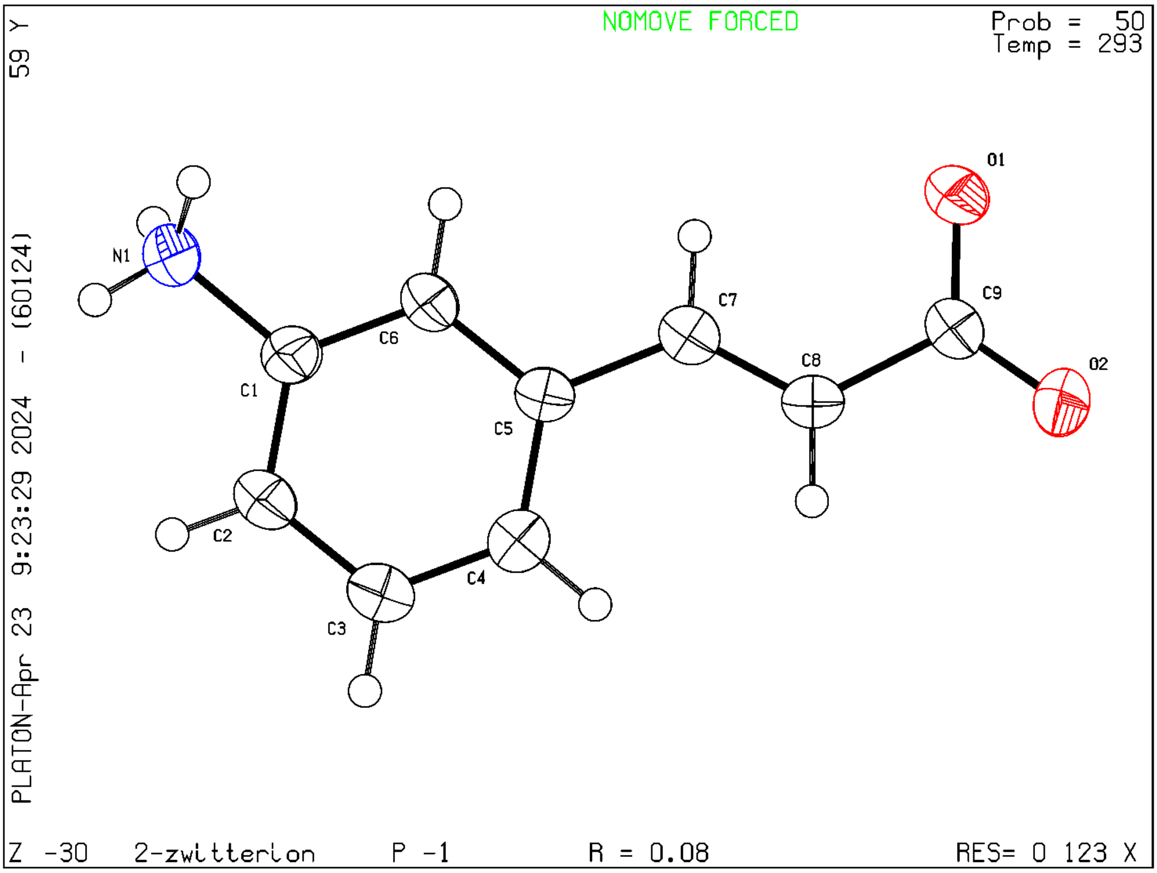

Datablock Zn-0D - ellipsoid plot

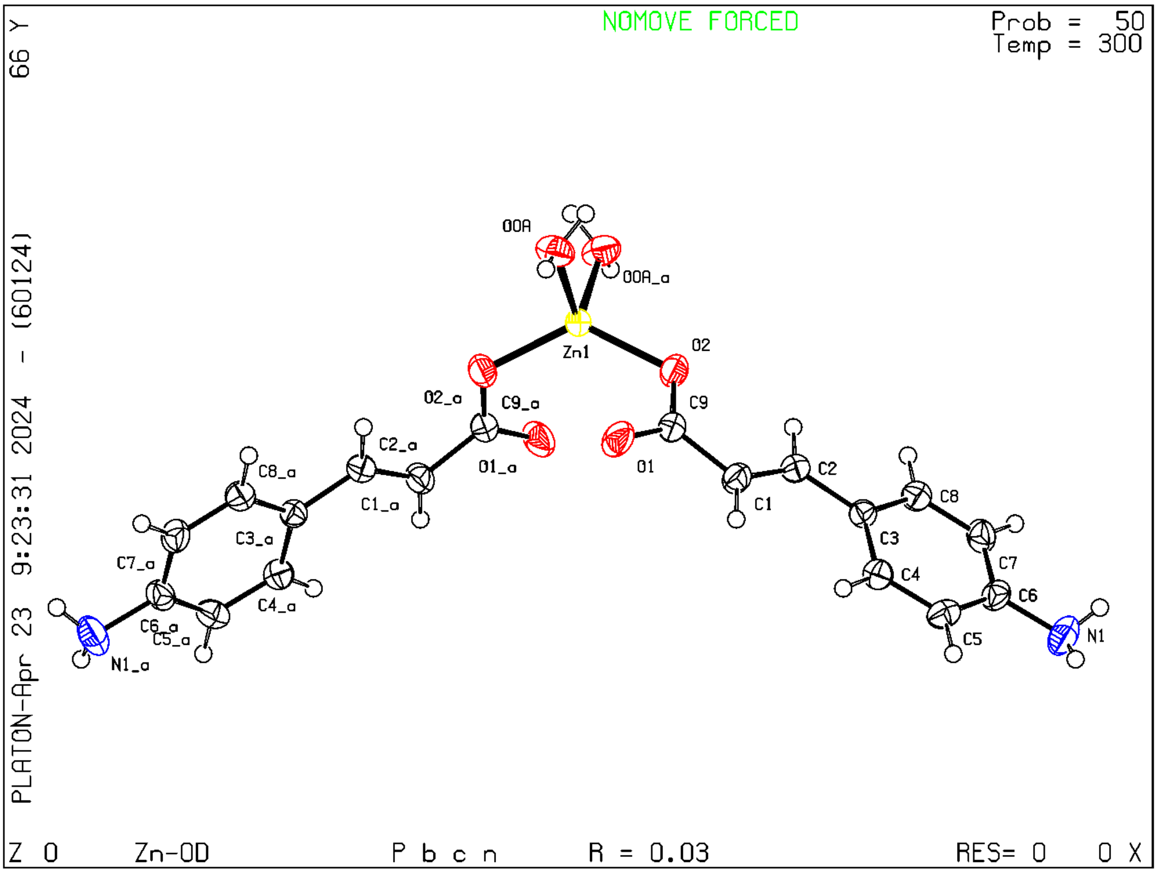

Datablock Zn-2D - ellipsoid plot

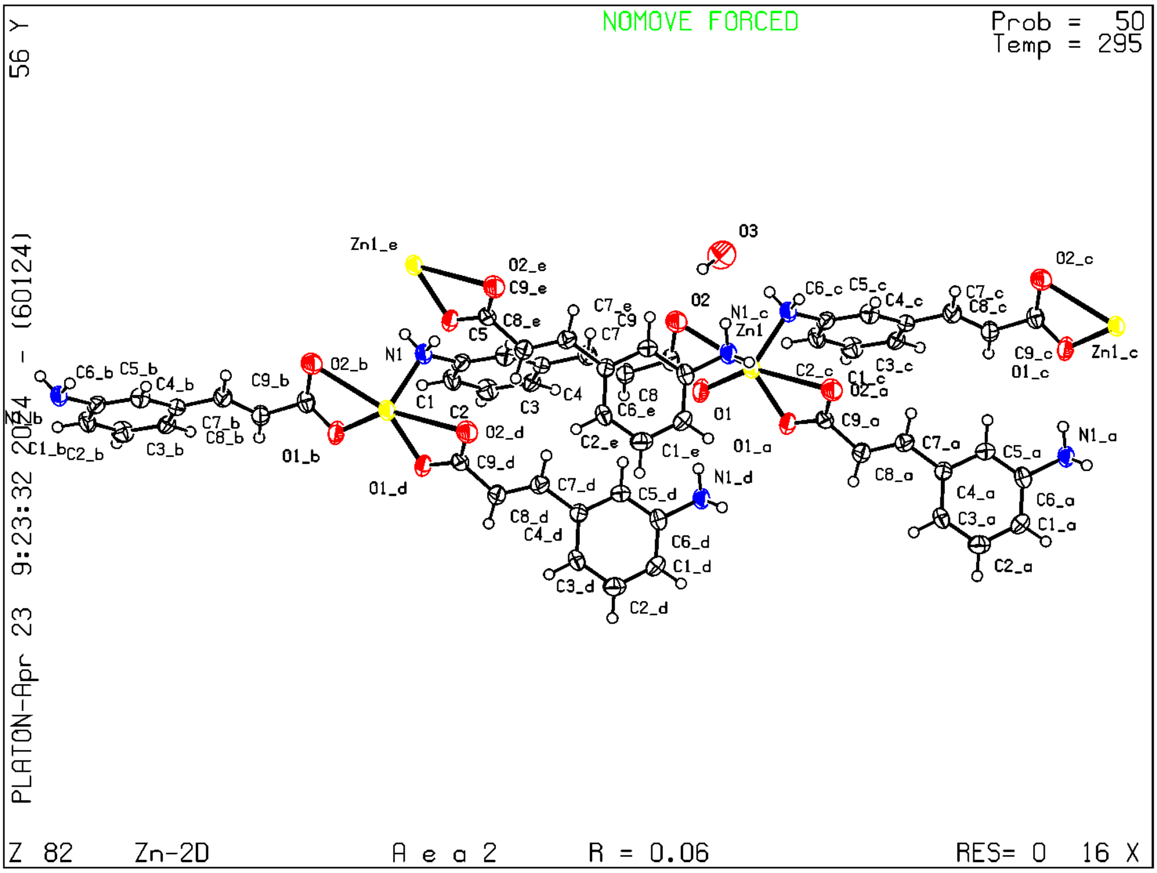

Datablock Zn-3D - ellipsoid plot

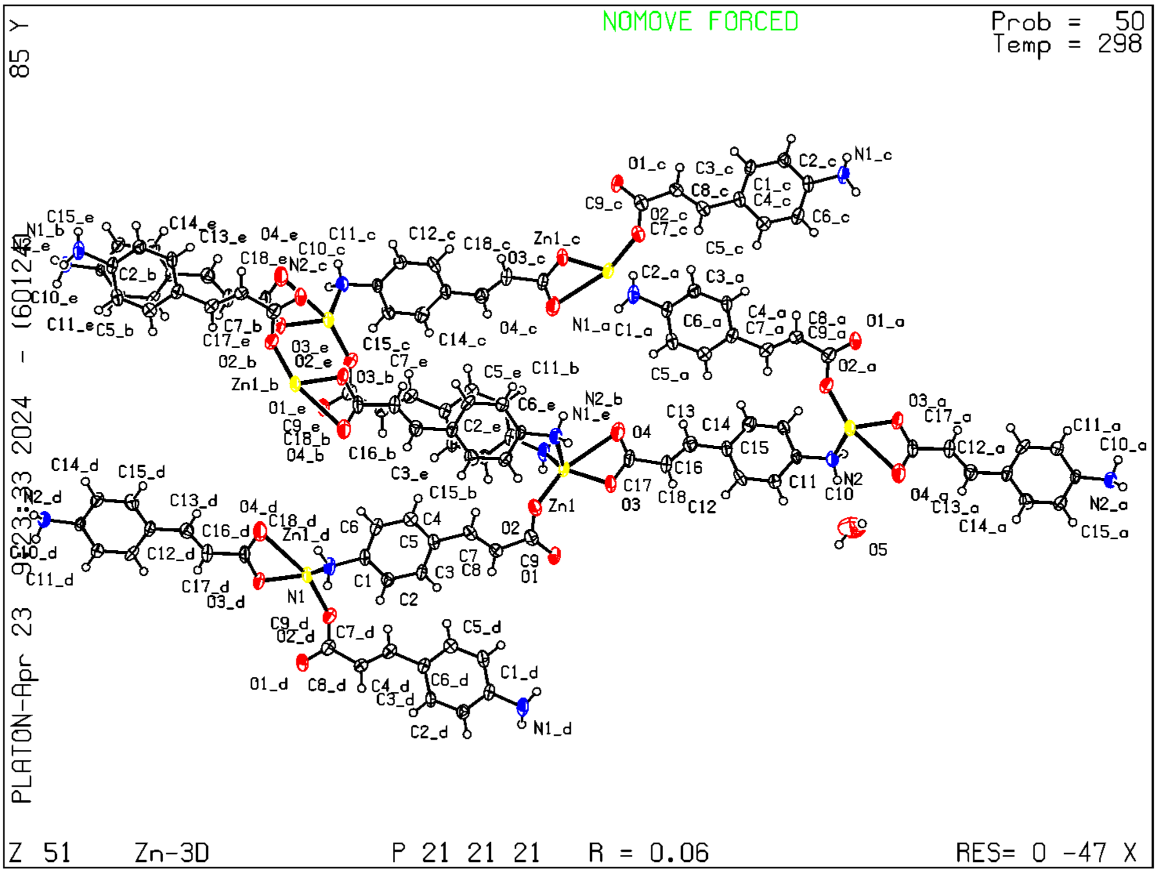

Supplement: Supplementary file 1 [file DataSheet1.PDF]
