# Supplementary material for: From 0D-complex to 3D-MOF: changing the antimicrobial activity of zinc(II) via reaction with aminocinnamic acids
Source: Front Chem. 2024 Jul 8;12:1430457. doi: 10.3389/fchem.2024.1430457 (PMC11260639; doi:10.3389/fchem.2024.1430457)
Supplement: Supplementary file 2 [file DataSheet2.docx]

**From 0D-complex to 3D-MOF: Changing the antimicrobial activity of zinc(II) *via* reaction with aminocinnamic acids**

**Simone d’Agostino,^1^ Laura Macchietti,^1^ Raymond J. Turner,^2^ and Fabrizia Grepioni^1^**

1 Dipartimento di Chimica “Giacomo Ciamician”, Università di Bologna, Via Selmi 2, 40126 Bologna, Italy.

2 Department of Biological Sciences, University of Calgary, Calgary, Alberta, Canada, T2N 1N4.

Supplementary Material

(7 pages)

| Crystal data and refinement details | page 2 |
| --- | --- |
| Crystal structure of 3-ACA | page 3 |
| Comparison between calculated and experimental diffraction patterns | page 4 |
| Rietveld refinement plot of Zn[(4-AC)(3-AC)] | page 5 |
| Thermogravimetric analyses | page 5 |
| ATR-FTIR spectra | Page 6 |
| Antimicrobial assays – Disk diffusion | Page 7 |
| Antimicrobial assays – Planktonic log kill and Biofilm % | Page 8 |

# Crystal data and refinement details

**Supplementary Table S1.** Crystal data and refinement details for 3-ACA (neutral and zwitterionic), [Zn(4-AC)_2_(H_2_O)_2_], [Zn(4-AC)_2_]∙H_2_O, [Zn(3-AC)_2_]∙2H_2_O, and [Zn(4-AC)(3-AC)] (powder data).

|  | **3-ACA Form I** | **3-ACA Form II**  **(zwitterion)** | **[Zn(4-AC)_2_(H_2_O)_2_]**  **0D complex** | **[Zn(4-AC)(3-AC)]**  **1D coord.polymer** | **[Zn(3-AC)_2_]∙2H_2_O**  **2D MOF** | **[Zn(4-AC)_2_]∙H_2_O**  **3D MOF** |
| --- | --- | --- | --- | --- | --- | --- |
| **Formula** | C_9_H_9_NO_2_ | C_9_H_9_NO_2_ | C_18_H_20_N_2_O_6_Zn | C_18_H_16_N_2_O_4_Zn | C_18_H_20_N_2_O_6_Zn | C_18_H_18_N_2_O_5_Zn |
| **FW (g/mol)** | 163.17 | 163.17 | 425.73 | 389.724 | 425.73 | 407.71 |
| **Crystal System** | Monoclinic | Triclinic | Orthorhombic | Triclinic | Orthorhombic | Orthorhombic |
| **Space Group** | P2_1_/n | P-1 | Pbcn | P-1 | Aba2 | P2_1_2_1_2_1_ |
| **a/Å** | 14.4183(17) | 4.2692(6) | 7.5978(2) | 7.7277(11) | 17.1162(14) | 7.7157(6) |
| **b/Å** | 5.0308(6) | 9.7343(13) | 11.4111(3) | 10.9627(16) | 21.0447(11) | 10.1066(7) |
| **c/Å** | 23.208(3) | 10.1777(14) | 20.8074(6) | 11.2913(17) | 5.1540(3) | 21.9887(12) |
| **α/°** | 90 | 111.122(3) | 90 | 65.411(3) | 90 | 90 |
| **β/°** | 102.107(11) | 90.504(11) | 90 | 71.035(3) | 90 | 90 |
| **γ/°** | 90 | 90.661(11) | 90 | 71.468(3) | 90 | 90 |
| **Volume/Å^3^** | 1645.9(3) | 394.48(10) | 1803.99(8) | 803.8(2) | 1856.5(2) | 1714.7(2) |
| **Z** | 8 | 2 | 4 | 2 | 4 | 4 |
| **T/K** | 298 | 298 | 298 | 298 | 298 | 298 |
| **ρ_calc_ g/cm^3^** | 1.317 | 1.374 | 1.567 | 1.610 | 1.523 | 1.579 |
| **μ/mm^-1^** | 0.094 | 0.098 | 1.4 | 2.354 | 1.360 | 1.465 |
| **measd rflns** | 6890 | 3012 | 14239 | - | 3762 | 5066 |
| **indep rflns** | 3766 | 1826 | 2289 | - | 2021 | 3366 |
| **R_1_** | 0.0757 | 0.0769 | 0.0322 | - | 0.0590 | 0.0572 |
| **wR_2_** | 0.1893 | 0.2100 | 0.0871 | - | 0.0914 | 0.0911 |
| **R_wp_** | - | - | - | 3.463 | - | - |

# Crystal structure of 3-ACA


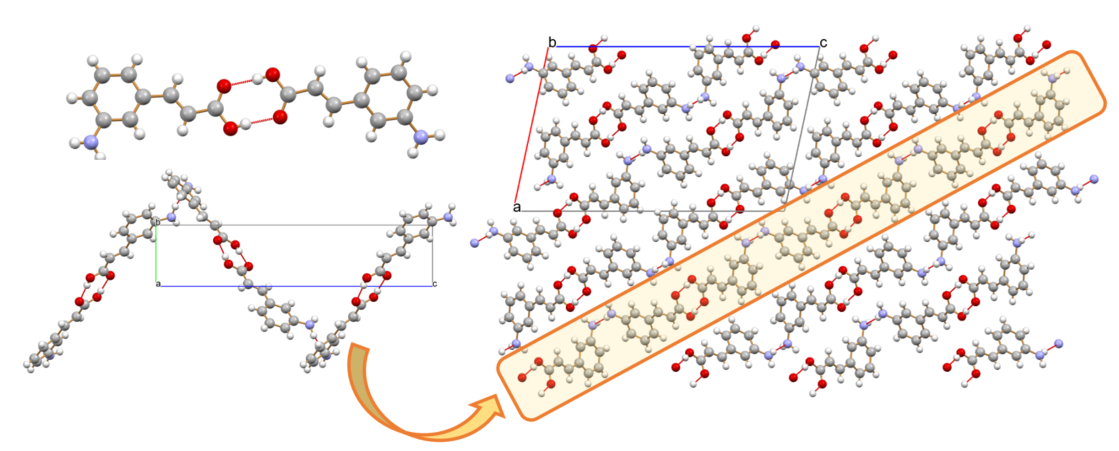


**Supplementary Figure S1.** Crystalline 3-ACA Form I: (top left) typical hydrogen bonding dimeric unit involving the carboxylic groups of two adjacent molecules; the dimers interact via -N(H)∙∙(H)N- hydrogen bonds in a chevron-like fashion (bottom left). Bottom right: a packing projection in the crystallographic *ac*-plane.


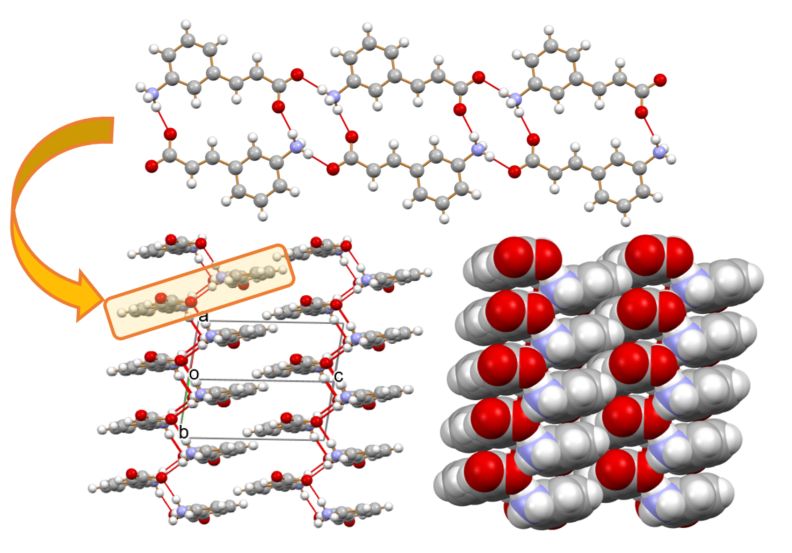


**Supplementary Figure S2.** Packing arrangement in crystalline 3-ACA Form II; each zwitterion is linked to the adjacent ones *via* hydrogen bonding rings between the carboxylate and ammonium groups, resulting in a 1D-ribbon (top). The ribbons are linked to each other via out-of-plane N(H)^+^∙∙∙^-^O(CO) hydrogen bonds (bottom left), and are stacked via π-π interactions along the 110 direction (bottom right).

# Comparison between calculated and experimental diffraction patterns

**Supplementary Figure S3.** Comparison between calculated (black) and experimental (blue) powder XRD patterns collected at RT for 4-AC and 3-AC. Note: in the case of 3-AC the experimental pattern fully matches the one calculated from Form I, while no trace of the zwitterionic Form II can be detected.

**Supplementary Figure S4.** Comparison between calculated (black) and experimental (blue) powder XRD patterns collected at RT for [Zn(4-AC)_2_(H_2_O)_2_], [Zn(4-AC)_2_]∙H_2_O, and [Zn(3-AC)_2_]∙2H_2_O; for the last two compounds the patterns (red) for the ball milling experiments are also reported.

# Rietveld refinement plot of Zn[(4-AC)(3-AC)]


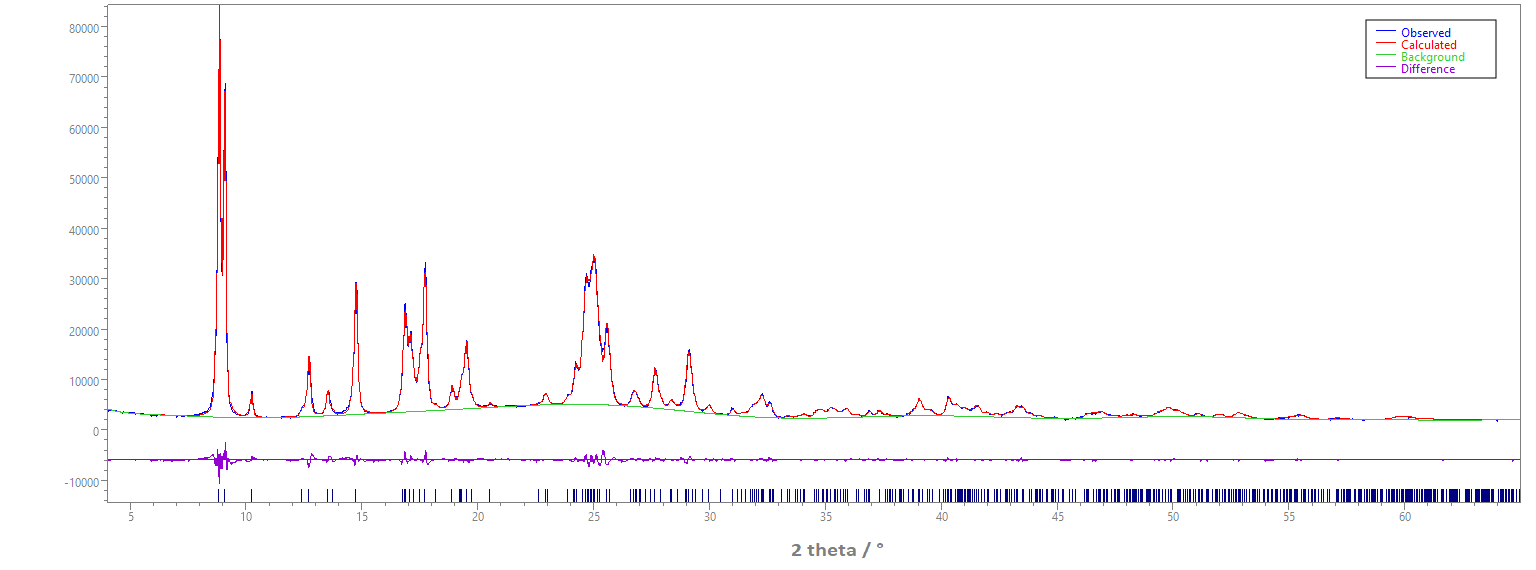


**Supplementary Figure S5.** Rietveld refinement for Zn[(4-AC)(3-AC)]: experimental (blue) and calculated (red) powder XRD patterns, and difference profile (magenta).

# Thermogravimetric analyses

**Supplementary Figure S6**. Thermogravimetric analyses of the compounds [Zn(4-AC)_2_(H_2_O)_2_] (black), [Zn(4-AC)_2_]∙H_2_O (red), [Zn(3-AC)_2_]∙2H_2_O (blue), and [Zn(4-AC)(3-AC)] (magenta).

# ATR-FTIR Spectra


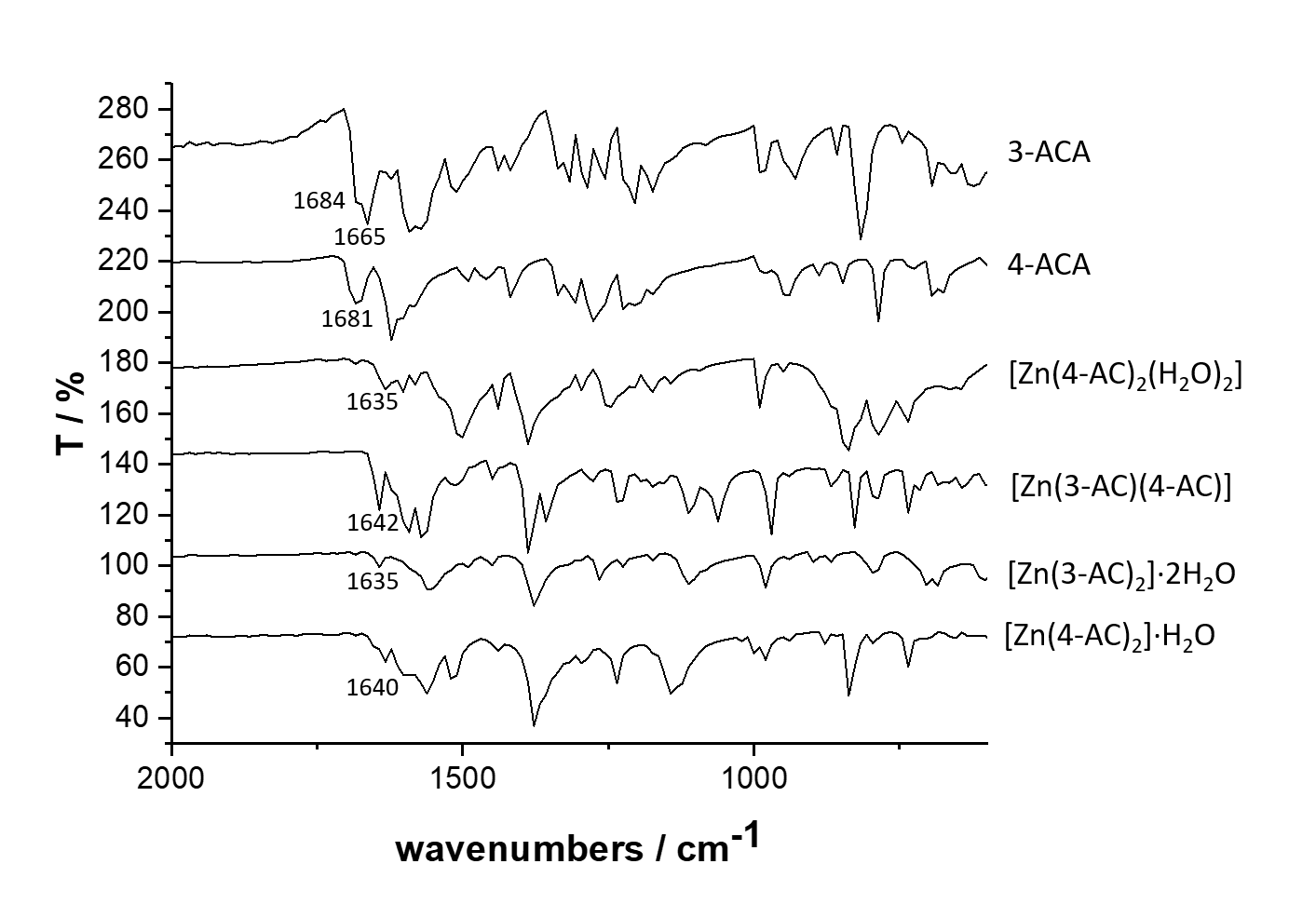


**Supplementary Figure S7**. ATR-FTIR spectra for polycrystalline samples of the ligands 3-ACA and 4-ACA, and of the products obtained from solution: [Zn(4-AC)_2_(H_2_O)_2_], [Zn(4-AC)(3-AC)], [Zn(3-AC)_2_]∙2H_2_O, and [Zn(4-AC)_2_]∙H_2_O.

# Antimicrobial assays – Disk diffusion


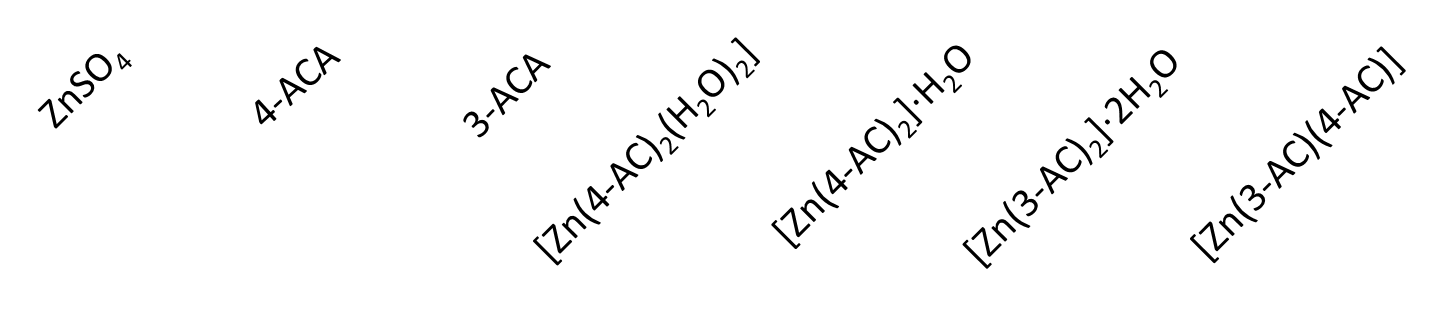


**Supplementary Figure S8**. Antimicrobial Activity of the compounds evaluated by Disk zone of inhibition. Larger zone of inhibition reflects ability of compound to associate then release from the filter disk and diffuse through the media to interact and inhibit growth of the bacteria (4 replicates).

# Antimicrobial assays – Planktonic log kill and Biofilm %


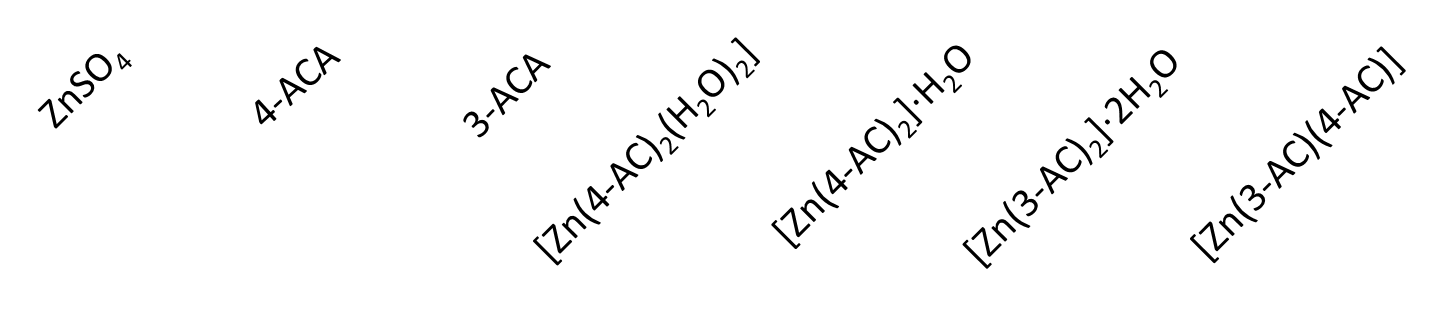


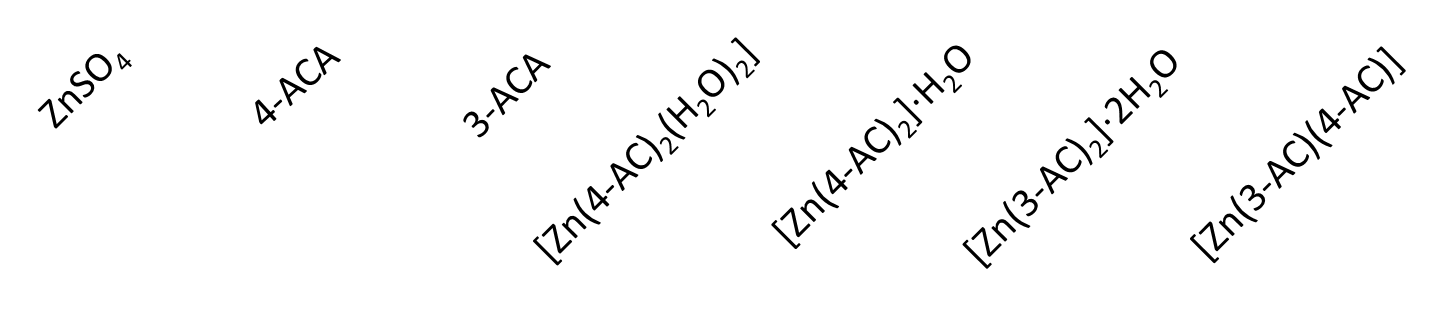


**Supplementary Figure S9**. Antimicrobial and antibiofilm activity of the compound. Concentration exposure at 2 mg/mL Zn equivalent levels). **A:** Planktonic cell kill reflecting the culture viable cell density reduction in log_10_. **B**: Evaluation of total biofilm density of cultures compared to unchallenged. (Averages and standard deviations from 4 replicates.)
